# Single-cell heterogeneity and cell-cycle-related viral gene bursts in the human leukaemia virus HTLV-1

**DOI:** 10.12688/wellcomeopenres.12469.2

**Published:** 2017-12-11

**Authors:** Martin R Billman, David Rueda, Charles R M Bangham

**Affiliations:** 1 Section of Virology, Division of Infectious Diseases, Department of Medicine, Imperial College London, Norfolk Place, London, UK; 2 Single Molecule Imaging Group, MRC London Institute of Medical Sciences, Du Cane Road, London, UK; 3 Section of Virology, Division of Infectious Diseases, Department of Medicine, Imperial College London, Hammersmith Hospital, Du Cane Road, London , UK

**Keywords:** retrovirus, latency, transcription, HTLV-, gene burs, RNA-FISH, smFISH

## Abstract

**Background**: The human leukaemia virus HTLV-1 expresses essential accessory genes that manipulate the expression, splicing and transport of viral mRNAs.  Two of these genes, *tax* and *hbz*, also promote proliferation of the infected cell, and both genes are thought to contribute to oncogenesis in adult T-cell leukaemia/lymphoma.  The regulation of HTLV-1 proviral latency is not understood. *tax,* on the proviral plus strand, is usually silent in freshly-isolated cells, whereas the minus-strand-encoded *hbz* gene is persistently expressed at a low level.  However, the persistently activated host immune response to Tax indicates frequent expression of *tax* *in vivo*.

**Methods**: We used single-molecule RNA-FISH to quantify the expression of HTLV-1 transcripts at the single-cell level in a total of >19,000 cells from five T-cell clones, naturally infected with HTLV-1, isolated by limiting dilution from peripheral blood of HTLV-1-infected subjects.

**Results**: We found strong heterogeneity both within and between clones in the expression of the proviral plus-strand (detected by hybridization to the *tax* gene) and the minus-strand ( *hbz *gene). Both genes are transcribed in bursts; *tax* expression is enhanced in the absence of *hbz*, while *hbz* expression increased in cells with high *tax* expression. Surprisingly, we found that *hbz* expression is strongly associated with the S and G _2_/M phases of the cell cycle, independent of *tax* expression.  Contrary to current belief, *hbz* is not expressed in all cells at all times, even within one clone.  In *hbz*-positive cells, the abundance of *hbz* transcripts showed a very strong positive linear correlation with nuclear volume.

**Conclusions**: The occurrence of intense, intermittent plus-strand gene bursts in independent primary HTLV-1-infected T-cell clones from unrelated individuals strongly suggests that the HTLV-1 plus-strand is expressed in bursts *in vivo*.  Our results offer an explanation for the paradoxical correlations observed between the host immune response and HTLV-1 transcription.

## Introduction

Human T-lymphotropic virus type 1 (HTLV-1) was the first human retrovirus to be discovered and infects approximately 10 million individuals around the world, causing an aggressive leukaemia or lymphoma or progressive lower limb weakness and paralysis in approximately 10% of infected individuals ^1^. Once infection is established in CD4 ^+^ and CD8 ^+^ T-lymphocytes, it persists lifelong in the host. The virus appears to be latent in the blood; however, the continuously activated anti-HTLV-1 immune response indicates frequent or persistent expression of HTLV-1 *in vivo* ^1– 3^. The regulation of HTLV-1 expression *in vivo* is not well understood.

In addition to the *gag*, *pol* and *env* genes common to all exogenous retroviruses, HTLV-1 encodes a *pX* region ^1^, which undergoes alternative splicing to express six accessory proteins that regulate transcription, splicing and transport of viral mRNAs. The accessory proteins also manipulate several key functions in the host cell. The two most important *pX* products are Tax, on the plus strand of the genome, and HBZ, the only gene encoded on the minus strand ^4, 5^. Several actions of Tax and HBZ are mutually antagonistic, but both Tax and HBZ play crucial roles in viral persistence, gene expression and leukaemogenesis ^5, 6^. Understanding how their expression is controlled is a key step towards understanding latency and expression of HTLV-1 in the host.

Earlier studies of HTLV-1 proviral expression have focused, at the cell population level, on detection either of protein ^2, 7, 8^ (e.g. by flow cytometry) or nucleic acid ^8, 9^ (e.g. by qPCR). Neither of these approaches is appropriate for HBZ, because it is expressed at a level near the limit of detection of current assays, including qPCR. However, the immune response to HBZ is an important correlate of the outcome of HTLV-1 infection ^10^. In addition, assays of viral expression in a cell population masks any heterogeneity of expression at the single-cell level. It is imperative to identify the extent and causes of such single-cell heterogeneity in order to understand the regulation of proviral latency.

We describe the use of single-molecule fluorescent *in situ* hybridisation (smFISH) to quantify the transcripts of plus-strand and *hbz* mRNA in individual cells of naturally-infected T-cell clones, isolated from patients' peripheral venous blood. We found that both the plus-strand and the minus-strand of the HTLV-1 provirus are expressed in intermittent bursts, with a surprising level of heterogeneity at the single-cell level in the expression of both the *hbz* gene and, especially, the plus-strand. The results reveal fundamental differences in the regulation of transcription of the provirus plus- and minus-strands, and suggest an explanation for the paradoxical differential effectiveness of the cytotoxic T-lymphocyte immune response to Tax and HBZ that is characteristic of HTLV-1 infection ^11^.

## Methods

### Derivation of T-lymphocyte clones from infected patients

Peripheral blood mononuclear cells (PBMCs) were isolated from the donated blood of HTLV-1+ patients, before individual clones were isolated and cultured as described in 12. Cells were distributed in 96-well plates at ^~^1 cell/well, using limiting dilution. The cells were then cultured with irradiated feeder cells, PHA, IL-2 and the retroviral integrase inhibitor raltegravir. Wells containing proliferating cells were tested for infection and proviral integrity using PCR. Linker-mediated PCR was then used as previously described to identify the proviral integration site and to verify that the population was indeed monoclonal ^13^.

The clones used, their integration sites and the patients they were derived from are summarised below:

**Table:** 

Clone	Patient	HTLV-1 Integration site
A	TBJ	Chr 4: 70,567,285
B	TBX	Chr 22: 44,323,168
C	TBW	Chr 19: 33,829,548
D	TBJ	Chr 14: 46,204,950
E	TCX	Chr 16: 53,601,059
F	TBW	Uninfected

### Cell culture, preparation and fixation

Patient-derived T-lymphocyte clones were cultured in RPMI-1640 medium (Sigma-Aldrich) with added L-glutamine (Invitrogen), penicillin and streptomycin (Invitrogen) and 10% AB human serum (Invitrogen) at 37°C, 5% CO _2_. IL-2 (Promokine) was added to the culture every 3 days, and the concentration of raltegravir (Selleck) was maintained throughout cell culture. In addition, the cells were activated every 14 days by the addition of beads coated with antibodies against CD2, CD3 and CD28 (Miltenyi-Biotech). All experiments were carried out on cells harvested on Day 8 of this cycle, after addition of fresh media on Day 7. Each clone was analysed in triplicate; cells from each triplicate sample were cultured separately for at least 24 hours before fixation.

Cells were added to glass coverslips (SLS, 12mm, number 1) coated with poly-L-lysine (Sigma-Aldrich), before being fixed in 2% formaldehyde (Life Technologies, in PBS) at room temperature for 15 minutes. Cells were then transferred to 70% ethanol for permeabilization or long-term storage at -20°C.

### Actinomycin D treatment

To quantify transcript half-life and intracellular distribution, and to investigate the link between bursts and transcription, cells were treated with actinomycin D (ActD) to block transcription. ActD (Sigma-Aldrich) was dissolved in DMSO (Sigma-Aldrich) at 1.5 mg/ml before being added to RPMI medium to a final concentration of 1.5 µg/ml. Two clones were tested, each in two biological replicates; DMSO alone was used as a loading control. After addition of ActD or DMSO, cells were fixed after 0, 2, 4, 6 and 22 hours of culture at 37°C before being stained, imaged and analysed.

### RNA-fluorescence *in-situ* hybridization (RNA-FISH)

*RNA-FISH* was carried out in accordance with the manufacturer’s protocols for the Stellaris probe system (Biosearch Technologies). Cells were permeabilised in 70% ethanol before being washed and incubated overnight at 37°C with the desired RNA-FISH probes (Stellaris). The cells were subsequently washed with DAPI (Sigma-Aldrich) and mounted in Vectashield mounting medium (Vectashield) on glass slides (Vector) and sealed with clear nail-varnish for imaging.

RNA-FISH probes were designed using Stellaris’s online probe design tool (version 4.1), with the reference genome AB513134 (Fan *et al.,* 2010 ^14^) used as input for the plus-strand probe (post-splice coding sequence) and *hbz* (post-splice coding sequence and 3’-UTR as reported in Kamentsky *et al.,* 2011 ^15^); the oligonucleotides used in each probe set are shown in Supplementary Figure 13. Probes targeting *hbz* were labelled with a Quasar-570 fluorophore and those targeting the plus-strand with a Quasar-670 fluorophore.

### Fluorescence microscopy

All coverslips were imaged using an Olympus IX70 inverted widefield microscope with a 100x-1.35NA UPlanApo oil objective and a Spectra Light Engine illumination source (Lumencor), with a resolution of 64 nm per pixel, and a spacing of 300 nm per optical slice. Z-offset was applied to correct for chromatic aberration, and exposures were adjusted for each respective wavelength to optimize the signal-noise ratio and minimize photobleaching. Excitation filters used were 390/18 (DAPI), 575/25 ( *hbz*-Quasar 570) and 632/22 (plus-strand-Quasar 670), with corresponding emission filters of 457/20, 632/60 and 692/40. A Coolsnap HQ camera (Roper Scientific) was used for initial images; the camera was later replaced with an ORCA-Flash 4.0 V2 digital CMOS camera (Hamamatsu).

### Image processing and analysis

After acquisition, all images passed through the following pipeline:

1. Visual inspection of image-stacks to ensure cells were captured in their entirety in the z-axis. Individual cells were excluded from the analysis. If more than a handful of cells were ‘cut-off’, the image was discarded.
2. Discarding of unneeded optical slices above and below cells in the image-stack, reducing image size to minimize subsequent processing and computing time.
3. Correction for uneven illumination. An illumination function was calculated using CellProfiler v2.2.0 ^15^, with a pipeline adapted from Roukos *et al.,* 2015 ^16^.

Images were then analysed with an adapted version of FISH-Quant, v3 ^17, 18^, run on MATLAB. The adaptation allowed step number 4, below:

1. Outlines marking cells and nuclei were automatically generated by thresholding and then manually double-checked.
2. All local maxima signals were characterised, to determine the signal intensity threshold which will best separate true and false positives, and calculate an optimal intensity threshold to define transcriptional ‘bursts’.
3. Spots passing intensity and shape thresholds were counted in each individual cell.
4. The stage of the cell-cycle was identified from the integrated intensity of each cell’s nuclear DAPI signal. While carried out in FISH-Quant, this process was adapted from the method described in Roukos *et al.,* 2015 ^16^. Cell-cycle gates were determined by visual inspection of the histograms, following the rule that cells in G _2_/M have double the DNA content (and therefore integrated intensity) of cells in G _0_/G _1_ ( Figure 5a; Supplementary Figure 15). Varying the width of the cell-cycle gates by 10% did not materially affect the conclusions.
5. The individual measurements – counts of putative single mRNA molecules, nascent ‘burst’ counts and cell cycle stage were collated and interpreted.

Finally, cells containing putative bursts were analysed individually using Imaris 3D-analysis software (Bitplane) to identify the 3D location of the burst relative to the ‘centre-of-gravity’ of the respective cell nucleus; the intranuclear location was then normalised by nuclear volume. Nuclear volume was estimated from the circle of best fit to the periphery of the DAPI staining, assuming a perfectly spherical nucleus.

### Statistical analysis

For each clone, three biological replicates were used, unless stated otherwise in figure legends. With exception of the logistic regression, all statistical analyses were carried out on Graphpad Prism 6, which was also used to create all graphs. Significance was symbolised in the following manner: ns (p > 0.05), * (p < 0.05), ** (p < 0.01), *** (p < 0.001) and **** (p < 0.0001).

In Figure 2, chi-squared tests were used to analyse burst frequency ( Figure 2c) and Mann-Whitney tests to analyse burst size and location ( Figure 2); all tests were two-tailed.

In Figure 3, Unpaired t-tests with Welch’s correction were used to analyse the changes in proportion of spots found within the nucleus ( Figure 3b). The half-life of *hbz* after ActD treatment was estimated using both a one-phase decay exponential fit of the raw total *hbz* per cell, and by linear regression of log (total cellular *hbz* per cell) against time ( Figure 3c).

The changes in frequency of plus-strand and *hbz* bursts shown in Figure 4 were all analysed using chi-squared tests, while the relationship between *hbz* and plus-strand expression and cell cycle stage was examined using binary and multinomial logistic regression with the “glm” and “multinom” functions respectively in R, v3.3.3 ^19^.

The expected frequency of *hbz*-negative cells in each clone was calculated from the observed mean *hbz* spot count in the respective clone ( Supplementary Table S1), using the Poisson distribution. The observed and expected numbers of *hbz*-positive and –negative cells were compared using chi-squared tests.

## Results

### smFISH reveals single-cell heterogeneity in HTLV-1 expression

**Figure 1.**
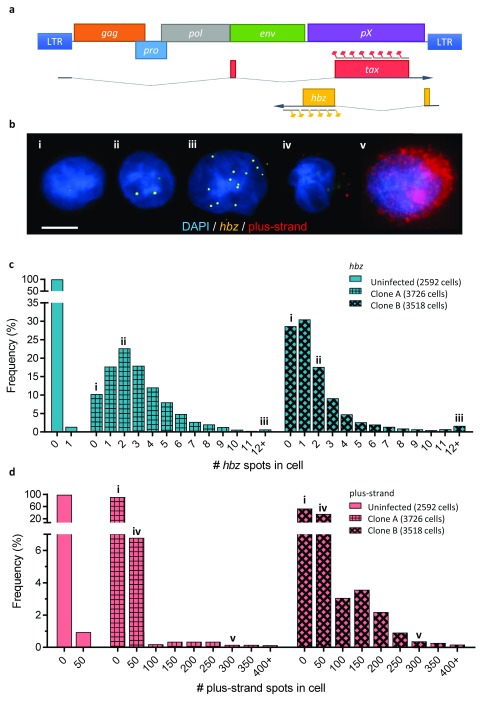
Single-molecule RNA-FISH reveals heterogeneity of plus-strand and *hbz* expression within and between clonal cell populations. **a**. Schematic of the HTLV-1 genome, showing the spliced transcripts of the plus-strand and *hbz*; the sequences targeted by probes on the plus-strand and the minus are marked by red and yellow flags respectively. **b**. Composite fluorescent micrograph with representative examples of cells expressing different levels of *hbz:* i) silent, ii) low (<5 spots) and iii) high (≥5 spots); and plus-strand RNA: iv) low (<100 spots), v) high (≥100 spots). The Roman numerals show the respective cells’ positions in panels **c** and **d**. Scale bar is 5 µm. **c**. Frequency distribution of *hbz* expression (number of *hbz* spots/cell) in individual cells, from one uninfected and two HTLV-1-infected clones. **d**. Frequency distribution of plus-strand expression in individual cells (same clones as in **c**); bins show the number of cells with 0-1 plus-strand spots (“0”), 2-50 spots (“50”), 51-100 spots (“100”), 101-150 spots (“150”) etc.

To quantify HTLV-1 expression at the single-cell level, and to identify any cell-to-cell heterogeneity that is lost in population-averaged approaches such as qPCR, we designed fluorescent-labelled probes to image mRNA from the plus-strand and the minus-strand ( *hbz*) of the provirus ( Figure 1a). The probe sequence we used to detect the plus-strand, in the pX region of the provirus, is present in all plus-strand transcripts. These probes were used to stain cells from five HTLV-1-infected T-cell clones, derived from patients’ PBMCs by limiting dilution; biological triplicates of each clone were studied in independent experiments. Each clone has a single, unique viral integration site ^12^; the provirus is complete in four clones, while in the fifth clone (Clone E) the 5’-LTR is deleted and the plus-strand thereby silenced, a phenomenon common in leukaemic clones ^20^. The diffraction-limited signal of a single spot in smFISH corresponds to a single mRNA molecule ^21^. In total, 19,477 individual cells were analysed, quantifying plus-strand and *hbz* transcripts and the proportion of transcripts within the nucleus, and identifying the cell cycle stage in each cell.

**Figure 2.**
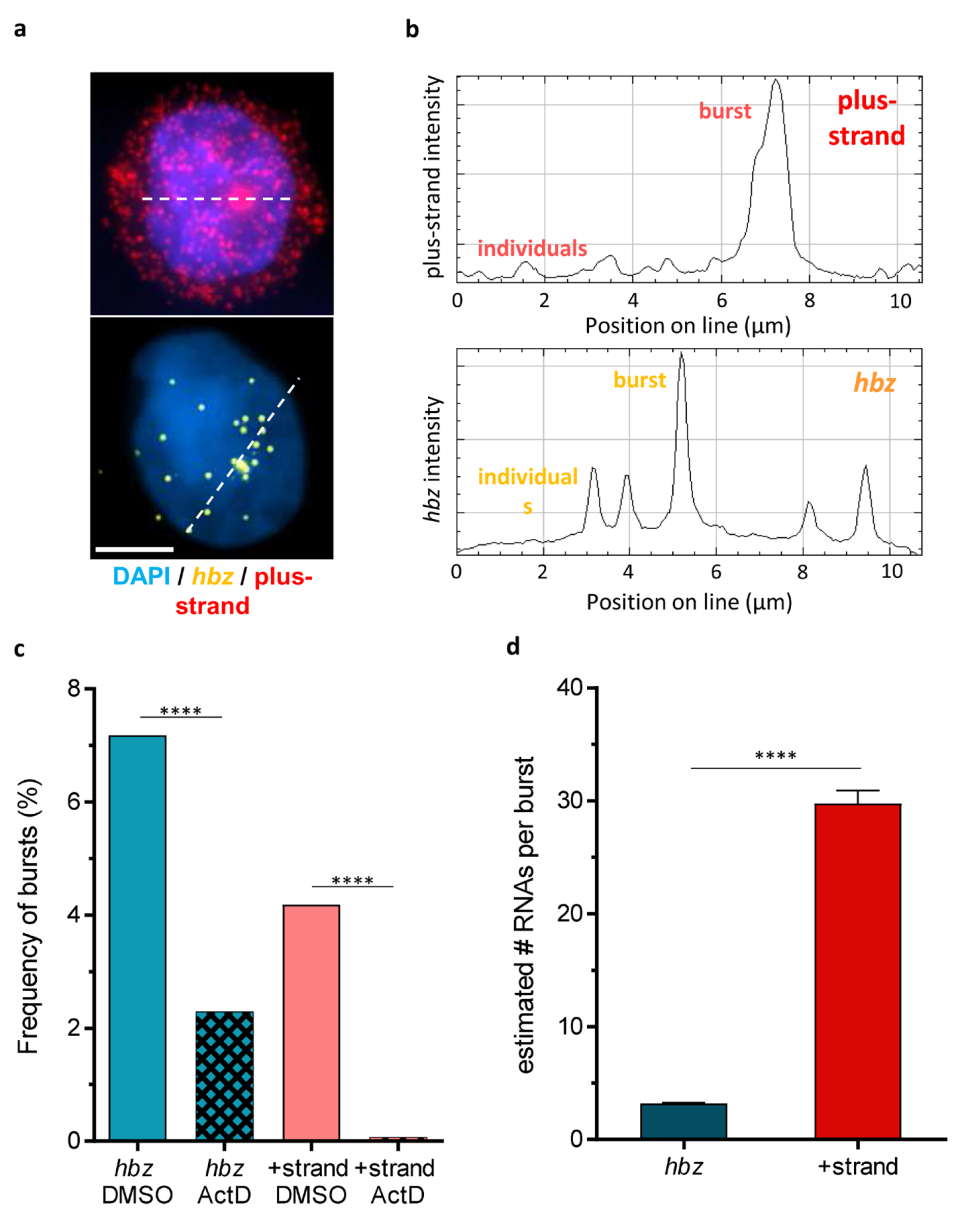
Both plus-strand and *hbz* are expressed in bursts; plus-strand bursts are much larger than *hbz* bursts. **a**. Representative plus-strand and *hbz* bursts. Dashed lines show the positions of the intensity profiles in panel b. Scale bar is 5 µm. **b**. Bursts are defined as specifically stained intranuclear spots that are significantly brighter than the main population of spots. **c**. Frequency of bursts is significantly reduced by blocking transcription with actinomycin D (n = 12,317 DMSO-treated cells, 7,120 actinomycin-D-treated cells from two replicates each of clones A and B). **d**. *hbz* bursts are small and uniform in size, whereas plus-strand bursts are significantly larger and more variable. SEM is shown, with 514 plus-strand and 800 *hbz* bursts from the pooled plus-strand-competent clones.

**Figure 3.**
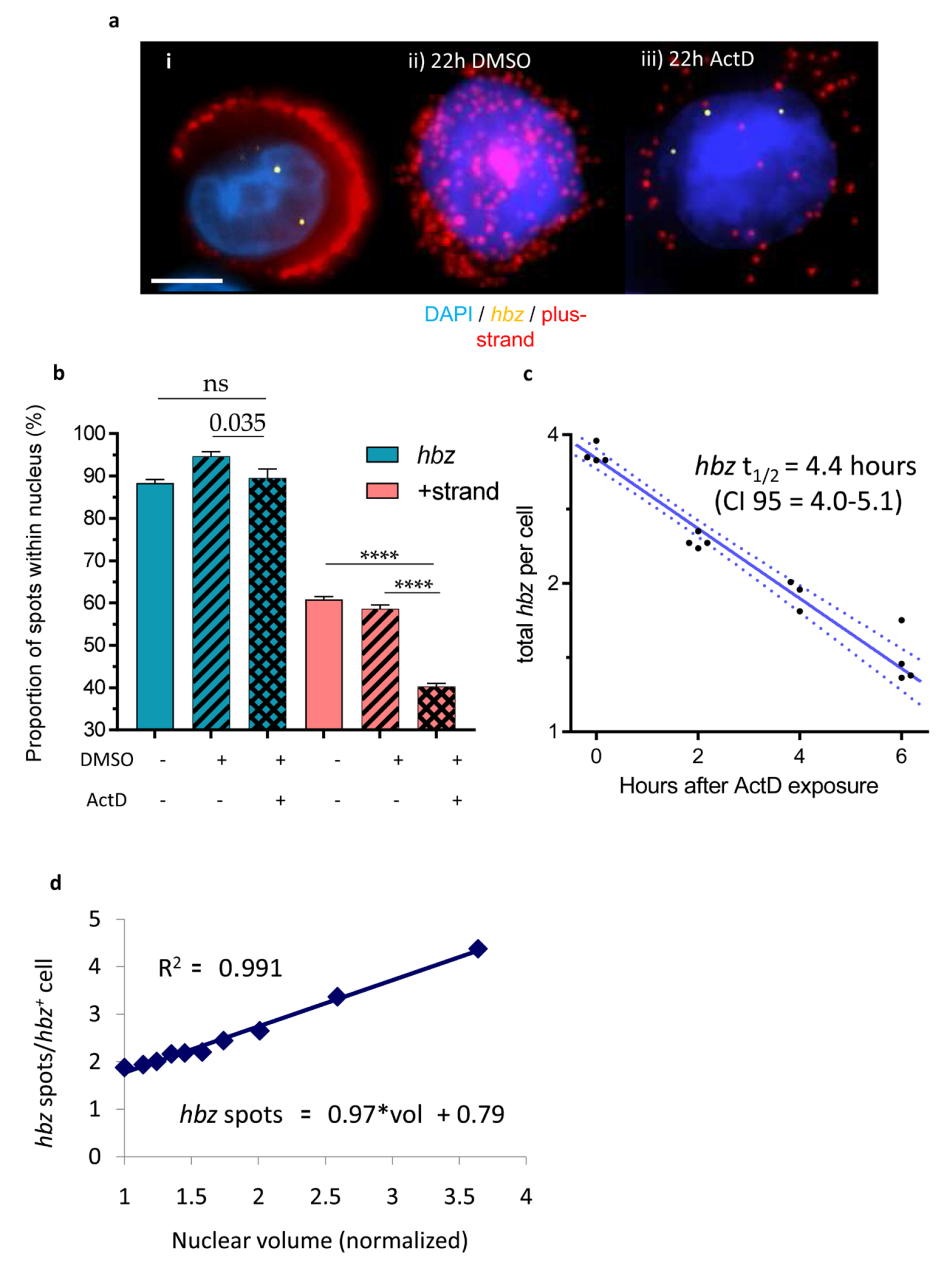
plus-strand RNA is exported from the nucleus, whereas the majority of *hbz* is retained. **a**. Representative images showing i) an optical slice illustrating the difference in cellular localisation of plus-strand RNA and *hbz*, ii) a typical plus-strand *-*high cell after 22 hours in DMSO and iii) a cell after 22 hours in actinomycin D. Image a.i is a single slice from a z-stack whereas ii and iii are maximum projections. Scale bar is 5 µm. **b**. *hbz* mRNA remains largely intranuclear, whereas plus-strand mRNA is mostly cytoplasmic; this difference is accentuated by blocking transcription with actinomycin D. n = 936 untreated, 390 DMSO-treated and 195 actinomycin D-treated cells, each from two pooled biological replicates of clones A and B. **c**. Rate of disappearance of total *hbz* spots following treatment with actinomycin D. The slope of the log-transformed data is the rate of decay. Individual measurements from replicates are shown, along with a linear regression and 95% confidence intervals (dashed lines); one of the four replicates failed at the 4 hour mark. n = 8126 total cells. **d**. Mean *hbz* spot count in *hbz*-positive cells was strongly correlated with nuclear volume.

The results show that *hbz* and the plus-strand have extremely different expression profiles ( Figure 1b). *hbz* levels were very low, as expected from previous observations in naturally-infected cells ^8^. A majority of cells were *hbz*+, but expression was not universal in any clone, ranging from 90% positive down to 65% ( Supplementary Figure 1). The *hbz*+ cells formed a unimodal population of low-expressing cells, most containing between 0 and 5 transcripts ( Figure 1b.ii). The frequency of cells with successively higher expression levels of *hbz* diminished rapidly: in ^~^20,000 cells, the highest observed *hbz* spot count was 25, although the range of expression varied between clones. The variation in total *hbz* mRNA between clones was explained by the variation in the proportion of *hbz*-expressing cells, not by differences in the mean level of *hbz* expression ( Figure 1c).

In contrast, the plus-strand presented a bimodal expression profile. In most clones, only a small fraction of cells were plus-strand+, but these cells expressed the plus-strand at a much greater level than *hbz*. Across all clones, cells with ‘high’ plus-strand expression (≥ 100 spots, Figure 1b.v) made up 4.9% of all cells, but contributed over 80% of all plus-strand mRNA detected. The variation in total plus-strand RNA between clones was due to the variation in the proportion of cells with this high level of expression ( Figure 1d).

Three of the clones studied (A-C) each had a similar range of intensity of expression. The maximum level of plus-strand RNA observed in clone D was much lower; however, all four clones had a bimodal distribution of plus-strand expression ( Supplementary Figure 2).

### HTLV-1 transcription occurs in bursts

In addition to the small proportion of cells with very high levels of expression, a characteristic feature of plus-strand expression was that many plus-strand+ cells also had a large nuclear spot which was typically much brighter than the average spot ( Figure 2a). We surmised that these bright spots corresponded to transcriptional bursts, which are increasingly recognized in mammalian genes ^22, 23^. In a transcriptional burst, transcripts are created faster than they diffuse away, and consequently they cannot be individually resolved. The resulting spot is larger and brighter than the average spot ( Figure 2b). The same characteristic spots were also identified for *hbz*, but with a smaller difference from average spots in intensity or size.

To test the hypothesis that the bright spots represent transcriptional bursts, we treated two of the clones (A and B) with ActD, to inhibit transcription and allow the putative bursts to disperse and disappear. Bursts were defined as intranuclear spots whose intensity significantly exceeded the main distribution of spot intensities ( Supplementary Figure 3). In 19,437 analysed cells, ActD treatment strongly and progressively reduced the frequency of these bright spots of both plus-strand and *hbz* ( Figure 2c). These observations are consistent with the view that the bright spots represent transcriptional bursts. A small proportion of the bright spots may be chance superpositions of multiple transcripts, but we conclude that a majority represent ongoing or recent transcription. Hereafter, we refer to these bright spots as bursts.

### Plus-strand bursts and *hbz* bursts differ in intensity

The burst sizes of the plus-strand and *hbz* were estimated by averaging thousands of individual diffraction-limited spots, which putatively represent single transcripts, and superposing their point-spread function (PSF) on that of the burst to estimate the number of transcripts present, given the 3D shape and intensity of that burst. Using this technique, we observed that *hbz* bursts were uniformly small in size, typically containing 3 to 4 spots, with very low cell-to-cell variation ( Figure 2d). Conversely, plus-strand bursts were much larger: the largest bursts were estimated to contain hundreds of transcripts. Plus-strand bursts were also more variable than *hbz* bursts in size (spot count). Bursts, whether plus-strand or *hbz*, were not uniformly distributed throughout the nucleus, and their spatial localization differed between clones ( Supplementary Figure 4).

### Plus-strand RNA is rapidly exported, *hbz* remains largely intranuclear

Tax protein generates a strong, persistently activated T-lymphocyte response in infected people ^10^. HBZ protein, in contrast, is expressed near the limit of detection of current methods, and T-cell receptor avidity for HBZ epitopes is correlated with both the proviral load and the disease outcome ^3, 10^. We therefore investigated the proportion of both transcripts that could be found in the nucleus or cytosol of cells.

Again, the differences between plus-strand RNA and *hbz* were stark. About 90% of all *hbz* spots were found in the nucleus, in each clone examined ( Figure 3b). This proportion did not change significantly after transcription had been blocked for 22 hours with ActD ( Figure 3a.iii).

In contrast, ~60% of plus-strand transcripts were found in the nucleus in 3 of 4 plus-strand-expressing clones. This percentage also fell after transcription was blocked ( Figure 3a,b). These estimates of the proportion of intranuclear mRNAs are not exact because they are derived from 2D maximum projections of the 3D data: spots lying above or below the nucleus along the z-axis are erroneously marked as intranuclear. This explains why the proportion of plus-strand RNA within the nucleus did not fall below 40% after 22 hr treatment with ActD, when 3D images indicate that the true proportion was lower. ( Figure 3a.i, iii). The fourth clone studied (clone D) was again an exception: in this clone, the proportion of plus-strand RNA within the nucleus was comparable to that of *hbz* ( Supplementary Figure 5).

### *hbz* mRNA abundance correlates with nuclear volume

The nuclear volume in each cell was estimated from the diameter of the DAPI staining in the maximum-projection image. There was a strong linear correlation between the nuclear volume and the number of *hbz* spots per cell ( Figure 3d). However, after taking *hbz* spot count into account, the frequency of *hbz* bursts was not correlated with nuclear volume ( Table S1d).

### *hbz* mRNA has a half-life of 4.4 hours

We estimated the half-life of *hbz* mRNA from the decline in *hbz* spot count during treatment with ActD. The high density of plus-strand spots in many plus-strand-expressing cells precluded a precise spot count of plus-strand RNA ( Supplementary Figure 14); as a result, it was not possible to make a reliable estimate of plus-strand RNA half-life.

Two biological replicates each of two separate clones were exposed to ActD (or DMSO as control) for 0, 2, 4 and 6 hours ( Supplementary Figure 6). A total of 15,524 cells were studied. The total counts of *hbz* spots, including the estimated sizes of bursts, were used to estimate the mean *hbz* content per cell; this mean value decreased over time ( Figure 3c), with a half-life of 4.4 hours (95% confidence interval of 5.1 hours to 4.1 hours).

### Transcription of the plus-strand is not independent of transcription of the minus-strand

Tax protein drives transcription of its own gene, in a positive feedback loop that is inhibited by HBZ protein ^24^. We therefore examined four plus-strand-expressing clones to test for a correlation between plus-strand and *hbz* expression.

**Figure 4.**
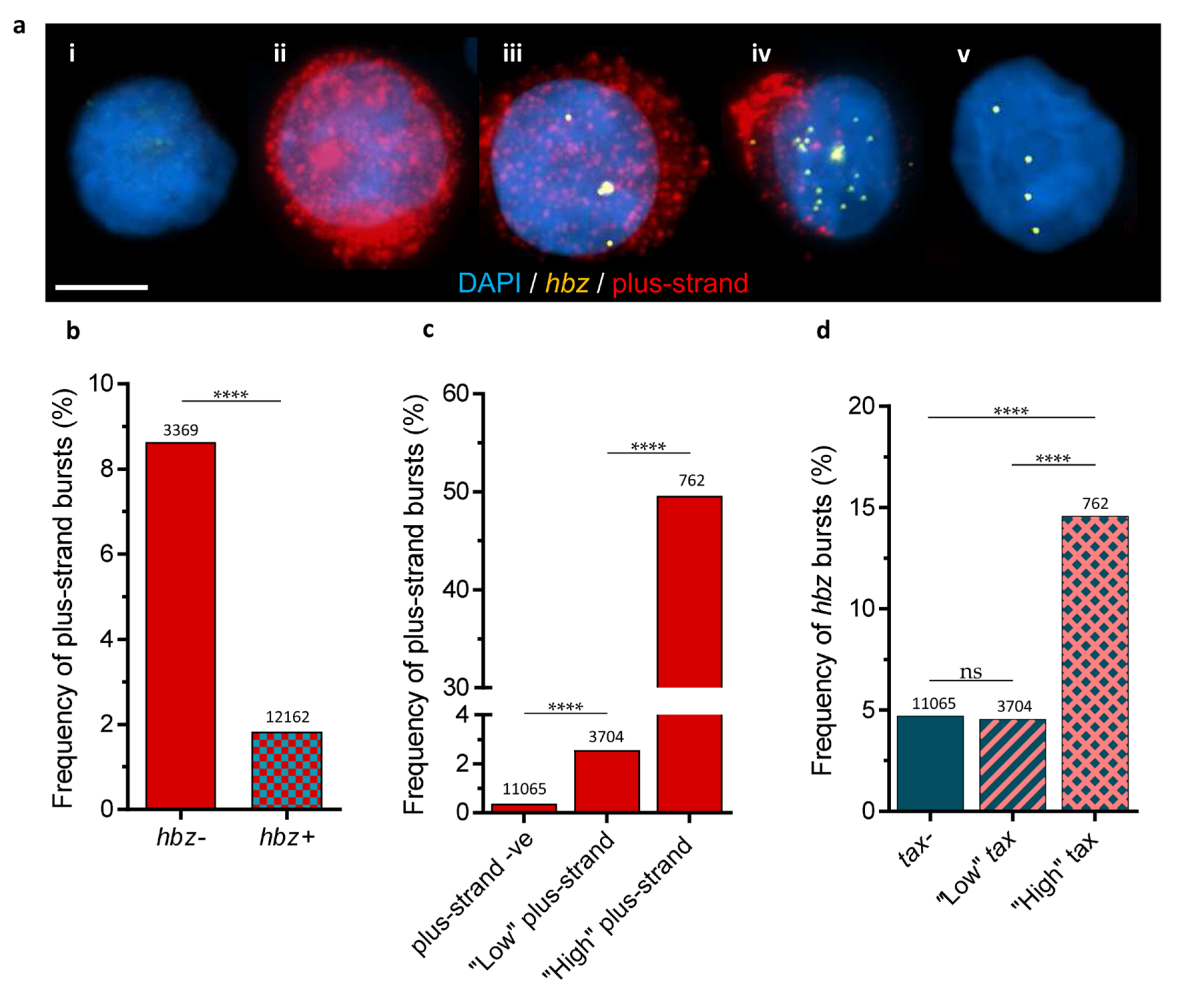
Relationship between expression of the plus-strand and *hbz* in individual cells. **a**. Representative images of a i) plus-strand *-*silent cell, ii) plus-strand-high cell (≥100 spots) with a plus-strand burst, iii) plus-strand-high cell without a plus-strand burst (note the presence of an *hbz* burst), iv) plus-strand-low cell (<100 spots) and v) plus-strand-silent cell, with low-level *hbz*. **b**. plus-strand bursts occur more frequently in *hbz*-negative cells. **c**. plus-strand bursts are indicative of very high plus-strand expression, with the proportion of cells which have a burst increasing as the level of plus-strand RNA increases. **d**. *hbz* bursts occur more frequently in high-plus-strand cells than in plus-strand-negative or plus-strand-low cells. Numbers above columns denote the number of cells in the corresponding population, from four pooled plus-strand-competent clones.

Plus-strand expression was strongly anti-correlated with the presence of *hbz*: cells were ~4 times more likely to have a plus-strand burst if they were *hbz*-negative ( Figure 4b); *hbz*-negative cells also had a higher average amount of plus-strand RNA. The count of plus-strand spots was positively correlated with the fraction of cells containing a plus-strand burst ( Figure 4c) in all clones studied, including clone D, although this clone differed from the other clones studied in having a lower peak plus-strand expression and a higher proportion of plus-strand mRNA retained in the nucleus.

Many of the cells with high plus-strand expression were *hbz*-negative ( Figure 4a.ii). However, bursts of *hbz* expression were almost three times more frequent in plus-strand-high cells than cells with no plus-strand or low levels of plus-strand RNA ( Figure 4a.iii, 4d). In fact, all of the 689 observed *hbz* bursts in low-plus-strand cells lacked a plus-strand burst ( Figure 4a.iv). This effect was observed in all three clones (A, B and C) that contained cells with high plus-strand expression. As further evidence of the reciprocal relationship between plus-strand and *hbz* expression, cells containing both a plus-strand burst and an *hbz* burst were significantly less frequent (2.9% of plus-strand-high cells) than expected by chance (7.2%); of the plus-strand-high cells, 14.6% had an *hbz* burst and 49.6% had a plus-strand burst (p < 0.0001; chi-squared). Finally, among high-plus-strand cells (N = 757), *hbz* bursts were significantly more frequent in cells without a plus-strand burst than in those with a plus-strand burst (23.2% vs. 5.8%; p < 10 ^-9^, chi-squared), consistent with the notion that the *hbz* burst inhibits plus-strand transcription ^24^ and thereby terminates the plus-strand burst.

These data ( Figure 4a) suggest a model for the temporal progression of HTLV-1 expression: see Discussion.

### *hbz* is not expressed in all cells at a given time

Since *hbz* is expressed at a very low level, the unexpectedly high observed frequency of *hbz*-negative cells might be due to a failure to detect *hbz* mRNA in some cells. If the *hbz* spots are distributed randomly among the cells in a clone, then the observed average frequency of ~2 to 3 *hbz* spots/cell would result in zero spots in ~5% to 18% of cells respectively (Poisson distribution; Supplementary Table S1). The observed frequency of *hbz*-negative cells was close to this random expectation (observed/expected = 1.1 and 1.2 respectively) in Clones D and E, but *hbz*-negative cells occurred at twice the expected frequency in Clones A, B and C (observed/expected = 1.9, 2.0 and 1.9 respectively) (p < 10 ^-38^ in each case; Supplementary Table S1). We note that the plus-strand is expressed at a high level in Clones A, B and C, but is low or absent in Clones D and E. These results show that the observed *hbz* spots were not randomly distributed among the cells, implying that some cells in each clone were not expressing *hbz* at a given instant.

### Cells in S phase and G _2_/M phase have elevated plus-strand and *hbz* expression

**Figure 5.**
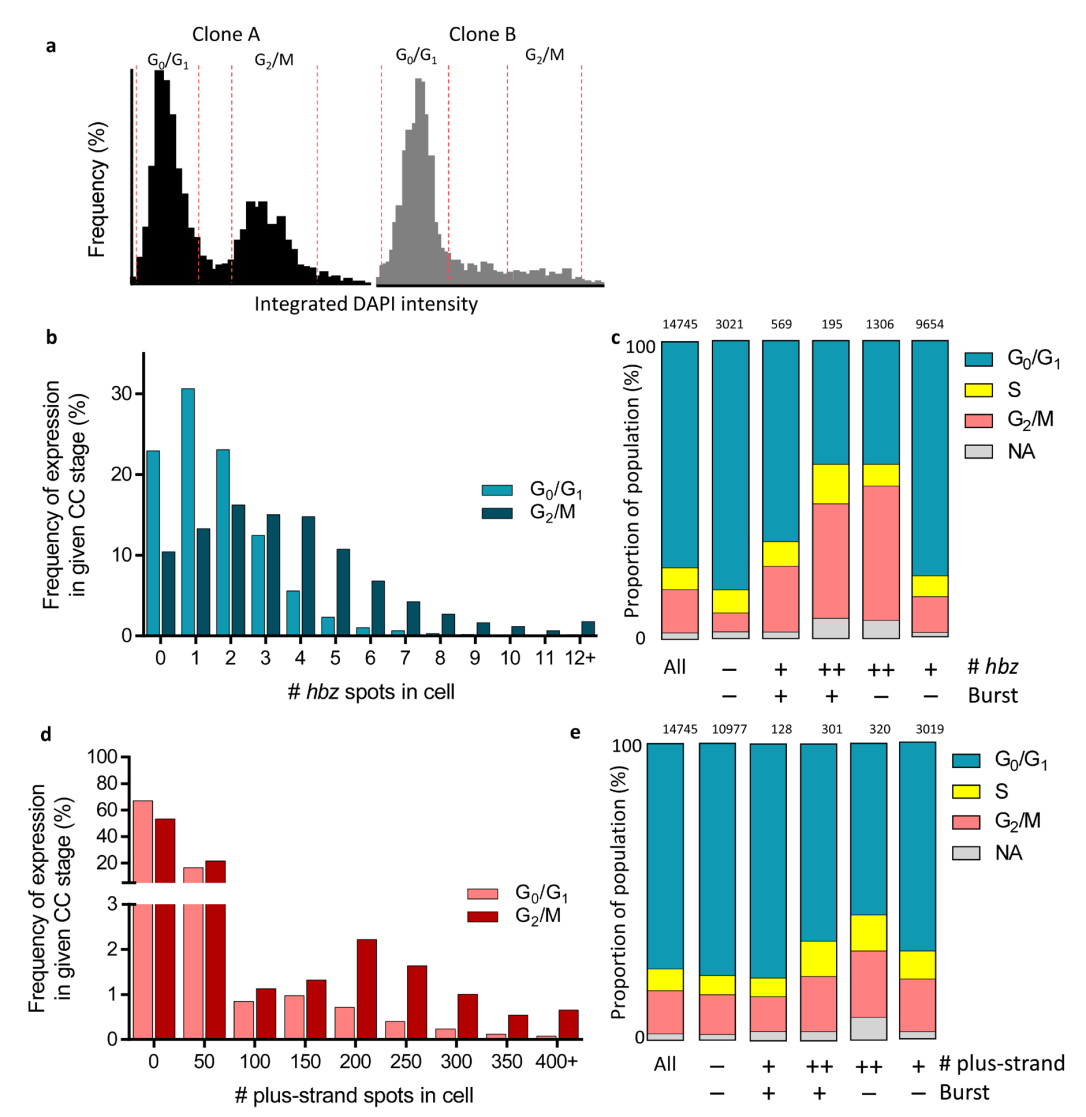
Expression of HTLV-1 plus-strand and *hbz* mRNAs varies with the stage of the cell cycle. **a**. Integrated intensity of DAPI in individual cells from replicates of two HTLV-1-infected T-cell clones. **b**. Cells in G _2_/M are more frequently *hbz*+ and express higher levels of *hbz* than do cells in G _0_/G _1_. **c**. Consistent with this observation, cells with high levels of *hbz* mRNA and/or an *hbz* burst are more likely to be found in S or G _2_/M (p < 0.0001, logistic regression analysis). **d**, **e**. Similarly, cells with high levels of plus-strand mRNA and/or a plus-strand burst are more likely to be found in S or G _2_/M. Cells are categorized according to the level of expression of mRNAs: – (1 or 0 plus-strand spots, 0 *hbz* spots), + (2-99 plus-strand spots, 1-4 *hbz* spots) and ++ (>99 plus-strand spots, >4 *hbz* spots), as well as by the presence or absence of bursts. “NA” denotes cells whose integrated nuclear intensity was too dim or bright to fit in one of the three cell cycle bins. Number of cells with a given level of viral expression stated above each bar. The four plus-strand *-*competent clones were pooled for this analysis, of which three had three biological replicate samples each, and one had two biological replicates; total n = 14,745 cells.

The cell-cycle stage of individual cells was identified by quantifying the integrated intensity of its DAPI-stained nucleus, which is linearly correlated with DNA content ^16^ ( Figure 5a). The cell-cycle profiles were reproducible between replicate samples of each respective clone ( Supplementary Figure 15), but there were marked differences between clones. Compared with cells in G _0_/G _1_, cells in G _2_/M showed a greater mean intensity (spot count) of expression of both *hbz* ( Figure 5b, Supplementary Figure 8) and the plus-strand ( Figure 5d, Supplementary Figure 9), and a higher frequency of *hbz* bursts. These effects were less pronounced in the plus-strand: only a small proportion of cells in G _2_/M were plus-strand-high ( Figure 5d). However, since plus-strand *-*high cells contribute over 80% of total plus-strand RNA, an increase in plus-strand-high cells from 2.6% in G _0_/G _1_ to 5.6% in G _2_/M resulted in a significant increase in total plus-strand expression. Plus-strand-silent and plus-strand-low cells were more likely to be in G _0/_G _1_, whereas plus-strand-high cells were more likely to be in S or G _2_/M ( Figure 5e), regardless of whether they contained plus-strand bursts. *hbz* expression showed a much stronger link with the cell cycle. Over half of all cells with high *hbz* ( Figure 5c) were in S or G _2_/M, as were one third of cells with low *hbz* and a burst.

Given that high Tax can lead to expression of *hbz* ^25^ the question arose whether *hbz* expression was independently correlated with the cell cycle, or correlated with plus-strand expression. Using logistic regression analysis, we found that both high plus-strand RNA and the cell cycle stage were independently correlated with *hbz* bursts ( Supplementary Figure 10, Supplementary Figure 11; p < 0.0001). Cells at a given stage of the cell cycle were on average 5.8 times more likely to have an *hbz* burst if they had high plus-strand expression than if they were plus-strand-low or silent, and a cell with a given level of plus-strand RNA was on average 50% more likely to contain an *hbz* burst if it was in G _2_/M rather than G _0_/G _1_ ( Supplementary Figure 12).

Both high *hbz* expression and an *hbz* burst were independently significantly associated with G _2_/M in all clones examined (p = 7.8 × 10 ^-27^ and p = 9.5 × 10 ^-4^, respectively; logistic regression analysis, Supplementary Figure 10, Supplementary Figure 11). Similarly, high plus-strand RNA was significantly correlated with G _2_/M in two of the three clones; the lack of significance in clone A may be attributable to sampling error on account of the rarity of plus-strand bursts in this clone. Although the level of plus-strand expression in Clone D was low as defined in this study, a higher plus-strand spot count in Clone D cells was still associated with S or G _2_/M.

## Discussion

In order for HTLV-1 to survive and propagate in the host, regulation of proviral latency is crucial. Three paradoxes regarding HTLV-1 proviral latency and the host immune response have remained unexplained. First, a strong cytotoxic T-lymphocyte (CTL) response to Tax protein is seen in all immunocompetent hosts, but the magnitude of this response does not explain the observed wide variation between individuals in the proviral load ^1, 11^. Second, the CTL response to HBZ is typically weak or undetectable, and *hbz* expression is low in polyclonal PBMCs, but the presence of a detectable anti-HBZ CTL response is associated with a lower PVL and a lower prevalence of inflammatory disease ^3, 10^. Third, the CTL response to Tax is chronically activated, implying frequent exposure to newly-synthesized Tax protein *in vivo*, but *tax* expression is usually undetectable in fresh PBMCs ^26^.

The results reported here offer an explanation for each of these paradoxes. By quantifying the frequency and intensity of *hbz* and plus-strand expression, we show that in clones of naturally-infected, CD4 ^+^ T-cells, both the plus-strand and minus-strand genes of HTLV-1 are expressed in bursts, which differ strongly in intensity and frequency between the two strands ( Figure 2). The sequence used here to detect the plus-strand of the provirus is present in all the viral plus-strand mRNAs. However, it is established that *tax* mRNA is the first and most abundant species ^26^: while some of the plus-strand signal detected in this study will represent the other plus-strand mRNA, species, the majority will be *tax* mRNA, especially in the early stages of the transcriptional burst.

If this pattern of HTLV-1 gene expression accurately represents the pattern of expression *in vivo*, the paradoxes can be explained as follows. At the single-cell level, expression of the plus strand is not uniform, but rather exhibits a bimodal expression profile ( Figure 1d). The minority of cells expressing plus-strand RNA at any one time express the transcripts at a very high level ( Figure 4c). The plus-strand transcripts are then rapidly exported to the cytosol ( Figure 3b). The resulting intermittent but intense expression of the highly immunogenic Tax protein is sufficient to account for the observed preponderance of anti-Tax CTLs ^27^. However, since the proportion of cells expressing Tax at any instant is low (of the order of 1% to 10%), the anti-Tax CTL response has a limited impact on the proviral load set-point.

In contrast, the proviral minus strand ( *hbz*) is expressed at a much lower level than the proviral plus strand, consistent with previous observations ^28, 29^, and much more uniformly across cells ( Figure 1c). However, *hbz* mRNA is less likely to be exported from the nucleus ( Figure 3b), again in agreement with earlier studies ^26^. This low expression combined with low translocation explains why HBZ protein levels are very low in physiological conditions. We have previously suggested ^26^ that HBZ protein expression is kept low in order to minimize exposure of the virus to the immune response; consistent with this hypothesis, HBZ protein is a weak T-cell immunogen ^30^. HBZ protein is frequently undetectable in fresh PBMCs, but Baratella et al. ^31^ reported the detection of HBZ protein in the cytoplasm of PBMCs isolated from patients with the inflammatory disease HAM/TSP. Our estimate of *hbz* half-life is higher than that made in previous studies ^32, 33^, but is set apart by using naturally-infected cells and absolute quantification.

Earlier investigations have pointed to an antagonistic relationship between Tax and HBZ: Tax can drive HBZ expression, which can then compete with Tax for the host factors which are necessary for expression driven from the promoter in the 5’-LTR ^24, 25^. These previous observations, in combination with our observations of the correlation between levels of plus-strand RNA and *hbz* within individual cells ( Figure 4), suggest the following model of the interaction between plus-strand expression and *hbz*. The plus-strand is more likely to be expressed in an *hbz*-silent cell ( Figure 4a.i), and once expressed the plus-strand typically reaches very high levels ( Figure 4.ii). A high level of plus-strand RNA, however, increases the probability of *hbz* expression ^25^ ( Figure 4iii), which in turn leads to cessation of plus-strand expression. Subsequently, both plus-strand and *hbz* transcripts decay, and the provirus remains latent until another factor upregulates expression, or the cell becomes silent, restarting the potential cycle. The factors that regulate the onset of plus-strand expression include glucose metabolism and the available molecular oxygen ^34^; cellular activation and stress may also contribute.

It has long been held that *hbz* is constitutively expressed in infected cells. However, all previous studies quantified HBZ expression in cell populations, using bulk-averaged techniques such as qPCR or antibody titres. Our single-molecule and single-cell approach indicates that *hbz* is not expressed constantly in all cells, even within a single clone. If the pattern of proviral expression we observed is also found *in vivo*, a temporary lapse in *hbz* expression could allow a plus-strand burst to develop, before plus-strand expression declines either under the influence of *hbz*, or immune detection and destruction. The pattern of frequent, low-level expression of *hbz* is consistent with the notion that the primary function of HBZ – at both protein and mRNA levels – is to maintain clonal persistence ^5, 6, 28, 35, 36^. Regardless of the cause, limiting expression of the highly immunogenic Tax protein to intermittent bursts allows the virus to optimise the protein’s effects in manipulating the host cell, to drive viral replication and clonal proliferation, while minimising its exposure to the intense anti-Tax immune response.

The cause of the observed cell-to-cell heterogeneity in *hbz* expression is unknown. It is also unclear precisely how the relationship between Tax and HBZ is mediated. The previously described mechanisms of interaction between Tax and HBZ act at the protein level ^37, 38^. However, given the very low proportion of *hbz* transcripts exported from the nucleus in any given cell and the low abundance of HBZ protein found in naturally-infected cells, it is possible that the *hbz* RNA plays a major part in this relationship. There was a very strong linear correlation between *hbz* spot count and nuclear volume ( Figure 3d). Certain cellular mRNAs show a similar dependence on nuclear volume or cell volume ^39^; the mechanisms responsible are not yet known. However, the frequency of *hbz* bursts was independent of nuclear volume, but was strongly associated with S/G _2_/M. This burst timing contrasts with the transcription rate of many cellular genes, which is reduced during S/G _2_/M, probably to compensate for the doubling of the gene copy number ^40^. The occurrence of an *hbz* burst in S/G _2_/M might confer two possible advantages on the virus. First, *hbz* promotes progression through the cell cycle ^41, 42^. Second, the burst will increase the abundance of both HBZ mRNA and protein molecules, which are normally present at limiting frequency, and thereby ensure efficient partitioning of the molecules between the two daughter cells ^43^.

**Figure 6.**
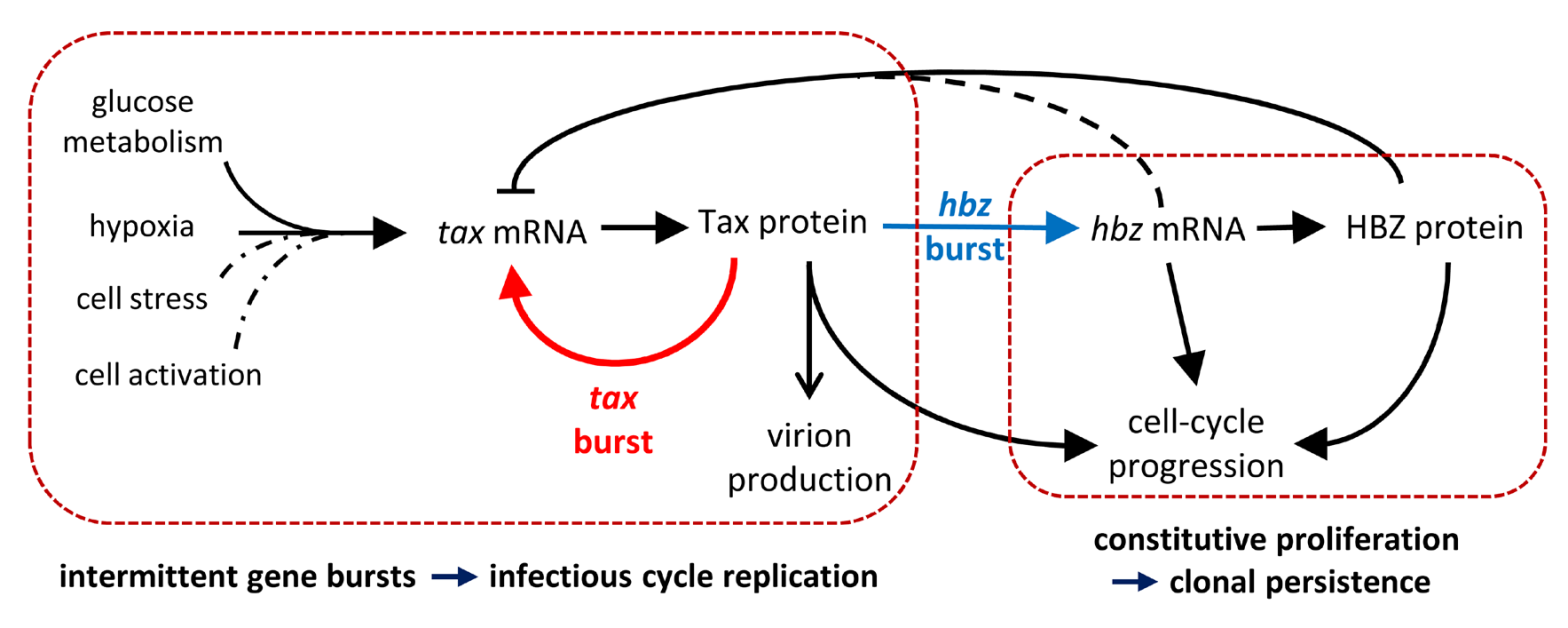
Model of regulation of HTLV-1 transcription and replication *in vivo*. Constitutive expression of *hbz* mRNA maintains clonal longevity by promoting cellular proliferation. Intermittent bursts of plus-strand expression, driven by bursts of *tax*, have three chief consequences: production of HTLV-1 virions and resulting infectious spread; accelerated cell-cycle progression; and stimulation of *hbz,* of which the RNA and protein both have proliferative effects. HBZ protein can also inhibit *tax* transcription, terminating the plus-strand burst. It is not known whether *hbz* mRNA also counteracts plus-strand transcription, but it is suggested by our results. The factors that regulate the frequency of plus-strand bursts include glucose metabolism and hypoxia; cell stress and cell activation may also trigger plus-strand expression. Aside from Tax, other triggers for *hbz* expression remain to be discovered. Dashed line represents a link indicated by results in the present study; dotted lines represent hypothetical links.

The observed longevity of HTLV-1-infected T-cell clones *in vivo* indicates that sustained cell proliferation plays a significant part in viral persistence ^13^. The relationship between the cell cycle and expression of the plus-strand and *hbz* is therefore of critical importance. We found that cells in G _2_/M had on average a significantly higher amount of plus-strand RNA, and the minority of cells with high plus-strand RNA were significantly more frequent in S or G _2_/M than in G _0_/G _1_ ( Figure 5). In contrast, plus-strand bursts were not significantly positively associated with S or G _2_/M phase ( Supplementary Figure 10), although the power of this test was limited by the low frequency and transient nature of plus-strand bursts. The strength of this relationship between plus-strand expression and the cell cycle varied between clones; in contrast, the relationship between *hbz* and the cell cycle was remarkably strong and consistent between clones. The logistic regression analysis indicated that each additional *hbz* transcript detected increased the odds of a cell being in G _2_/M (rather than G _0_/G _1_) by an average of 1.45-fold (7.3-fold in *hbz*-high cells), whereas each additional plus-strand transcript increased the odds by 1.004-fold (1.58-fold in plus-strand-high cells). This correlation with *hbz* was maintained in the clone incapable of expressing the plus-strand, showing that the relationship between *hbz* and the cell cycle is independent of plus-strand expression. Although several mechanisms have been described by which Tax protein and both HBZ protein and RNA promote cell proliferation ^35, 36, 41^, it remains possible that cell division itself enhances proviral expression: that is, proviral expression may be both cause and consequence of cell division. Since HTLV-1 persists in the host by driving proliferation and avoiding the immune response, it is a logical strategy to align its proviral expression with the host cell cycle, particularly for such an immunogenic product as Tax. We have summarised our current interpretation of the regulation of HTLV-1 transcription and replication in a model ( Figure 6) ^44– 46^.

Previous evidence suggested that *hbz* is constitutively expressed in HTLV-1-infected T-cells, albeit at a low level ^28, 35^. However, at a given time, *hbz* was not expressed in all cells in any of the clones studied here. The observed frequency of hbz-negative cells is inconsistent with a random (Poisson) distribution of *hbz* spots ( Supplementary Table S1); the departure from the Poisson distribution was consistently greater in the clones that expressed high levels of plus-strand RNA. We infer that *hbz* is not constitutively expressed in all cells. Either there is a fraction of cells within each clone that do not express *hbz* or, more likely, each cell passes through an *hbz*-negative phase, perhaps following a plus-strand burst. It is now important to identify the factors that trigger re-expression of *hbz*.

In summary, we show that plus-strand expression from the HTLV-1 provirus varies widely between individual cells, even within the same clone, and that minus-strand expression, while much more homogeneous than the plus-strand, is not present in all cells at all times. The contrast between the expression of the two strands is further demonstrated by difference between the frequency and intensity of their respective transcriptional bursts. Once expressed, the majority of plus-strand and *hbz* transcripts have different fates, with the majority of *hbz* retained in the nucleus while plus-strand RNA is exported to the cytosol. Our results also show that plus- and minus-strand expression do not occur independently of each other, but rather that, at the single-cell level, plus-strand expression is more likely to occur in the absence of *hbz*, whose expression is in turn more likely in cells with high plus-strand expression. Finally, proviral expression is correlated with the phase of the cell cycle, with clear implications for driving proliferation and evasion of immunosurveillance. It remains to be tested how closely the transcriptional behaviour of HTLV-1 observed in these naturally-infected T-cell clones represents the transcriptional behaviour of the virus *in vivo*, both qualitatively and quantitatively. We are now applying the techniques described here to quantify HTLV-1 plus- and minus-strand transcription in PBMCs both directly *ex vivo* and after short-term *in vitro* incubation. The ability to quantify these relationships at the single-cell level opens exciting avenues to elucidate the phenomenon of HTLV-1 latency.

## Ethical statement

HTLV-1-infected T-cell clones were derived from peripheral blood samples given by donors attending the National Centre for Human Retrovirology (NCHR) at Imperial College Healthcare NHS Trust, St Mary's Hospital, London. All donors gave written informed consent in accordance with the Declaration of Helsinki to donate blood samples to the Communicable Diseases Research Tissue Bank, approved by the UK National Research Ethics Service (15/SC/0089).

## Data availability

The data referenced by this article are under copyright with the following copyright statement: Copyright: © 2017 Billman MR et al.

Sample image data are available at: https://osf.io/b9mnd/. Further data are available on request from the authors; the large dataset (~2 Tb) is most reliably transferred on a hard disk.

## Supplementary material

**Supplementary File 1. Contains Supplementary Figures 1 to 15.**

**Supplementary Table S1. Analysis of frequency of *hbz*-negative cells in each of five HTLV-1-infected clones, A to E.**

## References

[1] Bangham CRM: Human T Cell Leukemia Virus Type 1: Persistence and Pathogenesis. *Annu Rev Immunol.* 2018;36:25–53. 10.1146/annurev-immunol-042617-053222 29144838

[2] Hanon E Hall S Taylor GP: Abundant tax protein expression in CD4+ T cells infected with human T-cell lymphotropic virus type I (HTLV-I) is prevented by cytotoxic T lymphocytes. *Blood.* 2000;95(4):1386–1392. 10666215

[3] Hilburn S Rowan A Demontis MA: *In vivo* expression of human T-lymphotropic virus type 1 basic leucine-zipper protein generates specific CD8+ and CD4+ T-lymphocyte responses that correlate with clinical outcome. *J Infect Dis.* 2011;203(4):529–536. 10.1093/infdis/jiq078 21208912 PMC3071236

[4] Gaudray G Gachon F Basbous J: The complementary strand of the human T-cell leukemia virus type 1 RNA genome encodes a bZIP transcription factor that down-regulates viral transcription. *J Virol.* 2002;76(24):12813–12822. 10.1128/JVI.76.24.12813-12822.2002 12438606 PMC136662

[5] Boxus M Willems L: Mechanisms of HTLV-1 persistence and transformation. *Br J Cancer.* 2009;101(9):1497–1501. 10.1038/sj.bjc.6605345 19861996 PMC2778510

[6] Ma G Yasunaga J Matsuoka M: Multifaceted functions and roles of HBZ in HTLV-1 pathogenesis. *Retrovirology.* 2016;13:16. 10.1186/s12977-016-0249-x 26979059 PMC4793531

[7] Satou Y Yasunaga J Zhao T: *HTLV-1 bZIP factor* induces T-cell lymphoma and systemic inflammation *in vivo*. *PLoS Pathog.* 2011;7(2):e1001274. 10.1371/journal.ppat.1001274 21347344 PMC3037353

[8] Shiohama Y Naito T Matsuzaki T: Absolute quantification of HTLV-1 basic leucine zipper factor (HBZ) protein and its plasma antibody in HTLV-1 infected individuals with different clinical status. *Retrovirology.* 2016;13:29. 10.1186/s12977-016-0263-z 27117327 PMC4847349

[9] Li M Kesic M Yin H: Kinetic analysis of human T-cell leukemia virus type 1 gene expression in cell culture and infected animals. *J Virol.* 2009;83(8):3788–3797. 10.1128/JVI.02315-08 19193802 PMC2663277

[10] MacNamara A Rowan A Hilburn S: HLA class I binding of HBZ determines outcome in HTLV-1 infection. *PLoS Pathog.* 2010;6(9):e1001117. 10.1371/journal.ppat.1001117 20886101 PMC2944806

[11] Kattan T MacNamara A Rowan AG: The avidity and lytic efficiency of the CTL response to HTLV-1. *J Immunol.* 2009;182(9):5723–5729. 10.4049/jimmunol.0900069 19380819

[12] Cook LB Rowan AG Melamed A: HTLV-1-infected T cells contain a single integrated provirus in natural infection. *Blood.* 2012;120(17):3488–3490. 10.1182/blood-2012-07-445593 22955925 PMC3482858

[13] Gillet NA Malani N Melamed A: The host genomic environment of the provirus determines the abundance of HTLV-1-infected T-cell clones. *Blood.* 2011;117(11):3113–3122. 10.1182/blood-2010-10-312926 21228324 PMC3062313

[14] Fan J Ma G Nosaka K: APOBEC3G generates nonsense mutations in human T-cell leukemia virus type 1 proviral genomes *in vivo*. *J Virol.* 2010;84(14):7278–7287. 10.1128/JVI.02239-09 20463074 PMC2898234

[15] Kamentsky L Jones TR Fraser A: Improved structure, function and compatibility for CellProfiler: modular high-throughput image analysis software. *Bioinformatics.* 2011;27(8):1179–1180. 10.1093/bioinformatics/btr095 21349861 PMC3072555

[16] Roukos V Pegoraro G Voss TC: Cell cycle staging of individual cells by fluorescence microscopy. *Nat Protoc.* 2015;10(2):334–348. 10.1038/nprot.2015.016 25633629 PMC6318798

[17] Mueller F Senecal A Tantale K: FISH-quant: automatic counting of transcripts in 3D FISH images. *Nat Methods.* 2013;10(4):277–278. 10.1038/nmeth.2406 23538861

[18] Tsanov N Samacoits A Chouaib R: smiFISH and FISH-quant - a flexible single RNA detection approach with super-resolution capability. *Nuc Acids Res.* 2016;44(22):e165. 10.1093/nar/gkw784 27599845 PMC5159540

[19] R Core Team: R: A Language and Environment for Statistical Computing. R Foundation for Statistical Computing, Vienna, Austria. R Foundation for Statistical Computing.2017.

[20] Takeda S Maeda M Morikawa S: Genetic and epigenetic inactivation of *tax* gene in adult T-cell leukemia cells. *Int J Cancer.* 2004;109(4):559–567. 10.1002/ijc.20007 14991578

[21] Raj A van den Bogaard P Rifkin SA: Imaging individual mRNA molecules using multiple singly labeled probes. *Nat Methods.* 2008;5(10):877–879. 10.1038/nmeth.1253 18806792 PMC3126653

[22] Raj A Peskin CS Tranchina D: Stochastic mRNA synthesis in mammalian cells. *PLoS Biol.* 2006;4(10):e309. 10.1371/journal.pbio.0040309 17048983 PMC1563489

[23] Tantale K Mueller F Kozulic-Pirher A: A single-molecule view of transcription reveals convoys of RNA polymerases and multi-scale bursting. *Nat Commun.* 2016;7:12248. 10.1038/ncomms12248 27461529 PMC4974459

[24] Lemasson I Lewis MR Polakowski N: Human T-cell leukemia virus type 1 (HTLV-1) bZIP protein interacts with the cellular transcription factor CREB to inhibit HTLV-1 transcription. *J Virol.* 2007;81(4):1543–1553. 10.1128/JVI.00480-06 17151132 PMC1797566

[25] Yoshida M Satou Y Yasunaga J: Transcriptional control of spliced and unspliced human T-cell leukemia virus type 1 bZIP factor (HBZ) gene. *J Virol.* 2008;82(19):9359–9368. 10.1128/JVI.00242-08 18653454 PMC2546946

[26] Rende F Cavallari I Corradin A: Kinetics and intracellular compartmentalization of HTLV-1 gene expression: nuclear retention of *HBZ* mRNAs. *Blood.* 2011;117(18):4855–4859. 10.1182/blood-2010-11-316463 21398577 PMC5292588

[27] Goon PK Biancardi A Fast N: Human T cell lymphotropic virus (HTLV) type-1-specific CD8 ^+^ T cells: frequency and immunodominance hierarchy. *J Infect Dis.* 2004;189(12):2294–2298. 10.1086/420832 15181578

[28] Saito M Matsuzaki T Satou Y: *In vivo* expression of the HBZ gene of HTLV-1 correlates with proviral load, inflammatory markers and disease severity in HTLV-1 associated myelopathy/tropical spastic paraparesis (HAM/TSP). *Retrovirol.* 2009;6:19. 10.1186/1742-4690-6-19 19228429 PMC2653460

[29] Satou Y Yasunaga J Yoshida M: *HTLV-1 basic leucine zipper factor* gene mRNA supports proliferation of adult T cell leukemia cells. *Proc Natl Acad Sci U S A.* 2006;103(3):720–725. 10.1073/pnas.0507631103 16407133 PMC1334651

[30] Rowan AG Suemori K Fujiwara H: Cytotoxic T lymphocyte lysis of HTLV-1 infected cells is limited by weak HBZ protein expression, but non-specifically enhanced on induction of Tax expression. *Retrovirology.* 2014;11:116. 10.1186/s12977-014-0116-6 25499803 PMC4282740

[31] Baratella M Forlani G Raval GU: Cytoplasmic Localization of HTLV-1 HBZ Protein: A Biomarker of HTLV-1-Associated Myelopathy/Tropical Spastic Paraparesis (HAM/TSP). *PLoS Negl Trop Dis.* 2017;11(1):e0005285. 10.1371/journal.pntd.0005285 28095504 PMC5271414

[32] Cavallari I Rende F Bona MK: Expression of Alternatively Spliced Human T-Cell Leukemia Virus Type 1 mRNAs Is Influenced by Mitosis and by a Novel *cis*-Acting Regulatory Sequence. *J Virol.* 2015;90(3):1486–1498. 10.1128/JVI.02298-15 26581997 PMC4719626

[33] Mocquet V Neusiedler J Rende F: The human T-lymphotropic virus type 1 tax protein inhibits nonsense-mediated mRNA decay by interacting with INT6/EIF3E and UPF1. *J Virol.* 2012;86(14):7530–7543. 10.1128/JVI.07021-11 22553336 PMC3416306

[34] Kulkarni A Mateus M Thinnes CC: Glucose Metabolism and Oxygen Availability Govern Reactivation of the Latent Human Retrovirus HTLV-1. *Cell Chem Biol.*in press,2017;24(11):1377–1387.e3. 10.1016/j.chembiol.2017.08.016 28965728 PMC5696563

[35] Satou Y Miyazato P Ishihara K: The retrovirus HTLV-1 inserts an ectopic CTCF-binding site into the human genome. *Proc Natl Acad Sci U S A.* 2016;113(11):3054–3059. 10.1073/pnas.1423199113 26929370 PMC4801255

[36] Matsuoka M Yasunaga JI: Human T-cell leukemia virus type 1: replication, proliferation and propagation by Tax and HTLV-1 bZIP factor. *Curr Opin Virol.* 2013;3(6):684–691. 10.1016/j.coviro.2013.08.010 24060211

[37] Basbous J Arpin C Gaudray G: The HBZ factor of human T-cell leukemia virus type I dimerizes with transcription factors JunB and c-Jun and modulates their transcriptional activity. *J Biol Chem.* 2003;278(44):43620–43627. 10.1074/jbc.M307275200 12937177

[38] Clerc I Polakowski N André-Arpin C: An interaction between the human T cell leukemia virus type 1 basic leucine zipper factor (HBZ) and the KIX domain of p300/CBP contributes to the down-regulation of tax-dependent viral transcription by HBZ. *J Biol Chem.* 2008;283(35):23903–23913. 10.1074/jbc.M803116200 18599479 PMC3259792

[39] Padovan-Merhar O Nair GP Biaesch AG: Single mammalian cells compensate for differences in cellular volume and DNA copy number through independent global transcriptional mechanisms. *Mol Cell.* 2015;58(2):339–352. 10.1016/j.molcel.2015.03.005 25866248 PMC4402149

[40] Skinner SO Xu H Nagarkar-Jaiswal S: Single-cell analysis of transcription kinetics across the cell cycle. *eLife.* 2016;5:e12175. 10.7554/eLife.12175 26824388 PMC4801054

[41] Mitobe Y Yasunaga J Furuta R: HTLV-1 bZIP Factor RNA and Protein Impart Distinct Functions on T-cell Proliferation and Survival. *Cancer Res.* 2015;75(19):4143–4152. 10.1158/0008-5472.CAN-15-0942 26383166

[42] Satou Y Yasunaga J Yoshida M: *HTLV-I basic leucine zipper factor* gene mRNA supports proliferation of adult T cell leukemia cells. *Proc Natl Acad Sci U S A.* 2006;103(3):720–725. 10.1073/pnas.0507631103 16407133 PMC1334651

[43] Soltani M Singh A: Effects of cell-cycle-dependent expression on random fluctuations in protein levels. *R Soc Open Sci.* 2016;3(12):160578. 10.1098/rsos.160578 28083102 PMC5210684

[44] Matsuoka M Jeang KT: Human T-cell leukemia virus type 1 (HTLV-1) and leukemic transformation: viral infectivity, Tax, HBZ and therapy. *Oncogene.* 2011;30(12):1379–1389. 10.1038/onc.2010.537 21119600 PMC3413891

[45] Overbaugh J Bangham CR: Selection forces and constraints on retroviral sequence variation. *Science.* 2001;292(5519):1106–1109. 10.1126/science.1059128 11352065

[46] Yoshida M: Multiple viral strategies of HTLV-1 for dysregulation of cell growth control. *Ann Rev Imm.* 2001;19:475–496. 10.1146/annurev.immunol.19.1.475 11244044

