# Supplementary material for: Single-cell heterogeneity and cell-cycle-related viral gene bursts in the human leukaemia virus HTLV-1
Source: Wellcome Open Res. 2017 Dec 11;2:87. Originally published 2017 Sep 22. [Version 2] doi: 10.12688/wellcomeopenres.12469.2 (PMC5645716; doi:10.12688/wellcomeopenres.12469.2)
Supplement: Supplementary file 1 [file wellcomeopenres-2-14485-s0000.tgz › 08cc5892-255d-4db8-9436-919b56539352.pptx]

## Slide 1
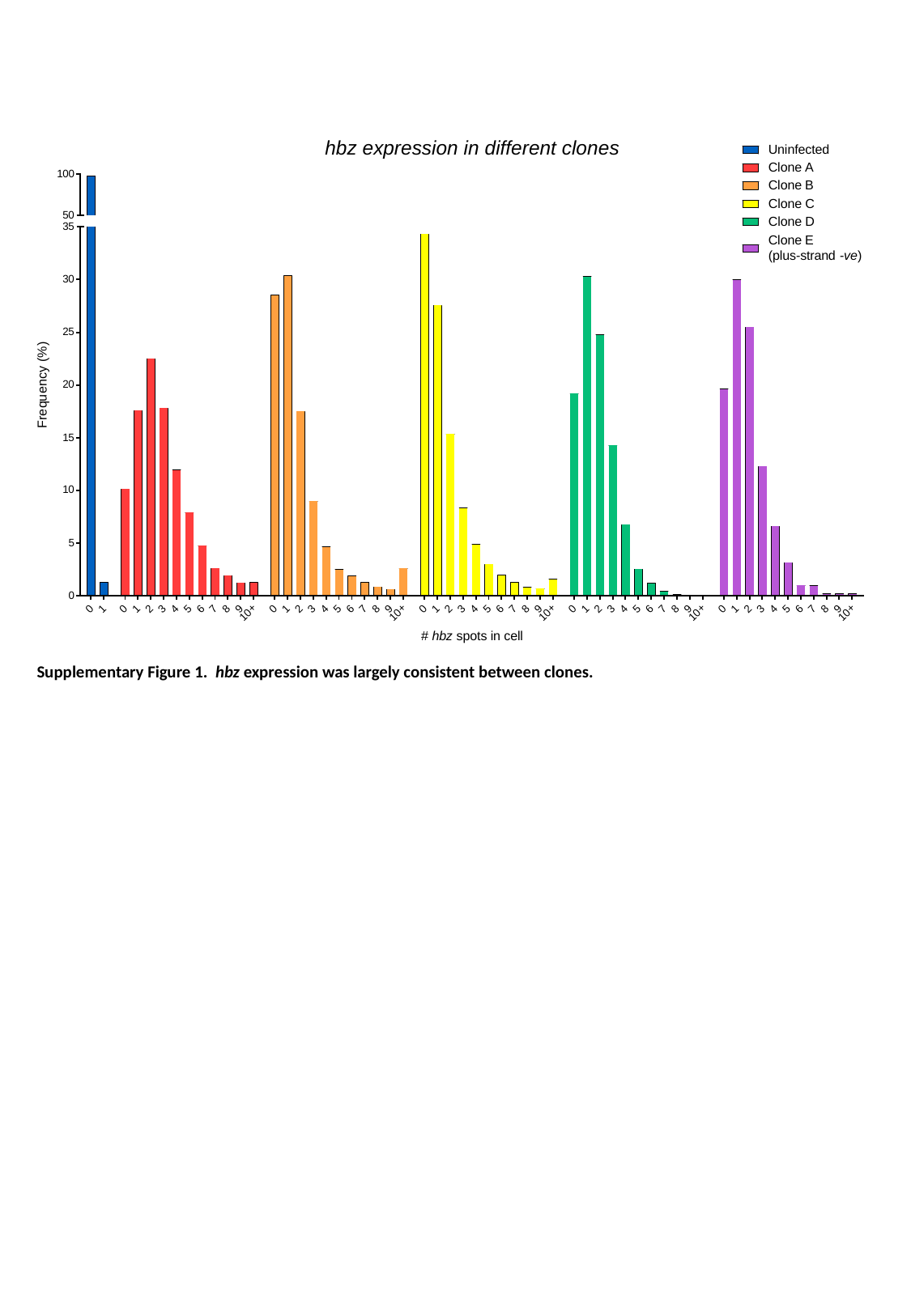

Supplementary Figure 1. hbz expression was largely consistent between clones.

## Slide 2
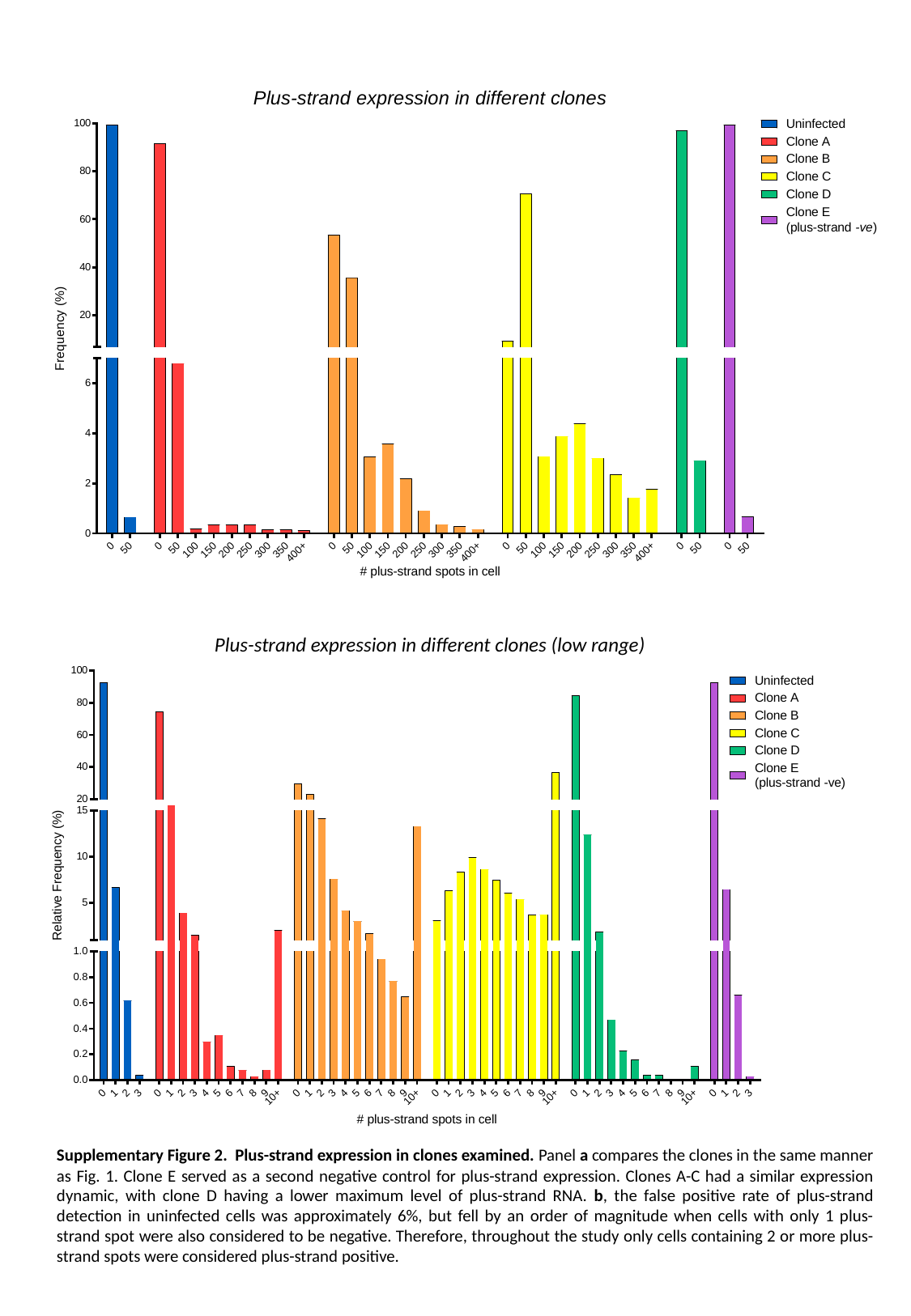

Plus-strand expression in different clones (low range)
Supplementary Figure 2. Plus-strand expression in clones examined. Panel a compares the clones in the same manner as Fig. 1. Clone E served as a second negative control for plus-strand expression. Clones A-C had a similar expression dynamic, with clone D having a lower maximum level of plus-strand RNA. b, the false positive rate of plus-strand detection in uninfected cells was approximately 6%, but fell by an order of magnitude when cells with only 1 plus-strand spot were also considered to be negative. Therefore, throughout the study only cells containing 2 or more plus-strand spots were considered plus-strand positive.

## Slide 3
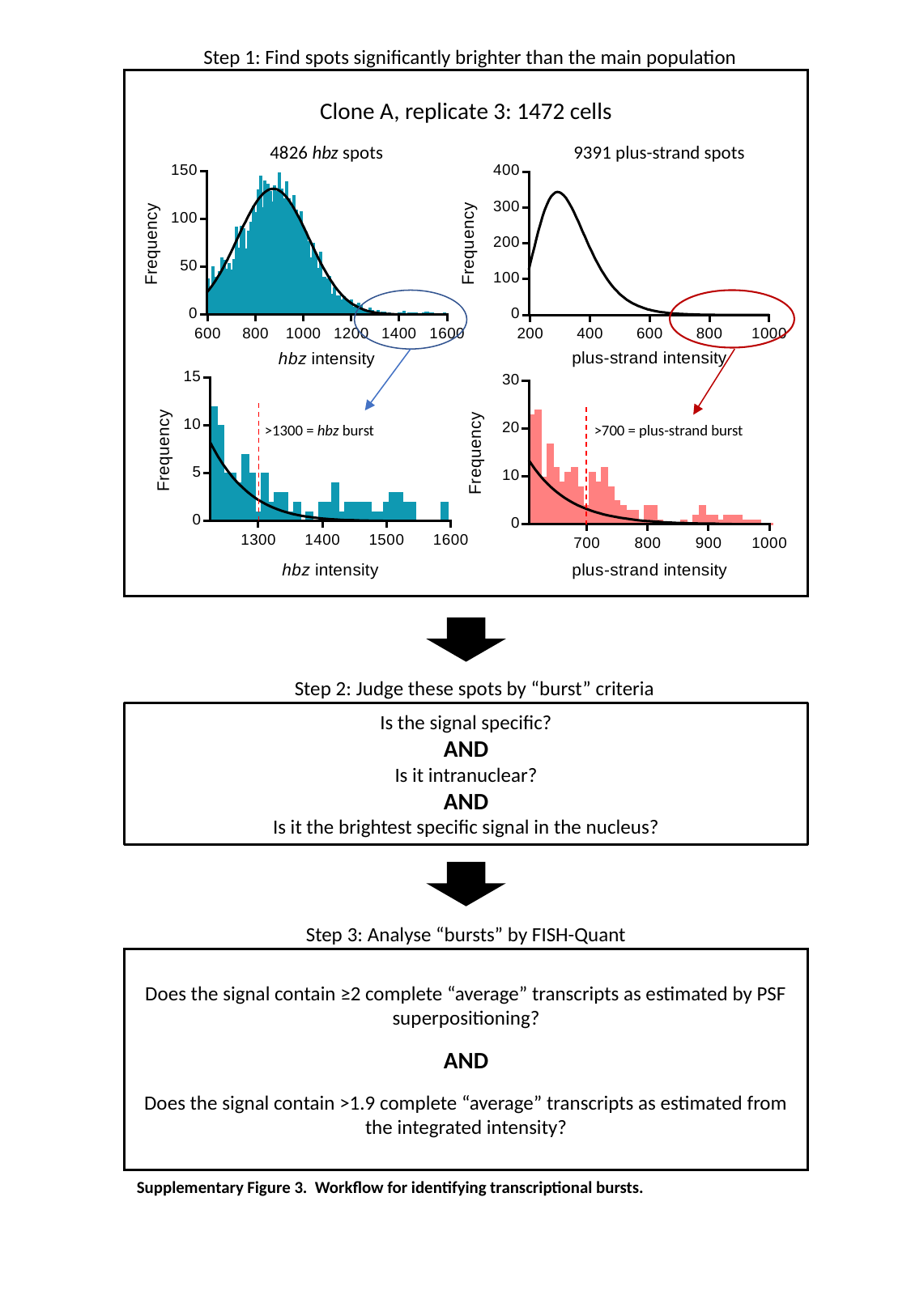

Step 1: Find spots significantly brighter than the main population
Clone A, replicate 3: 1472 cells
4826 hbz spots
9391 plus-strand spots
>1300 = hbz burst
>700 = plus-strand burst
Step 2: Judge these spots by “burst” criteria
Is the signal specific?
AND
Is it intranuclear?
AND
Is it the brightest specific signal in the nucleus?
Step 3: Analyse “bursts” by FISH-Quant
Does the signal contain ≥2 complete “average” transcripts as estimated by PSF superpositioning?
AND
Does the signal contain >1.9 complete “average” transcripts as estimated from the integrated intensity?
Supplementary Figure 3. Workflow for identifying transcriptional bursts.

## Slide 4
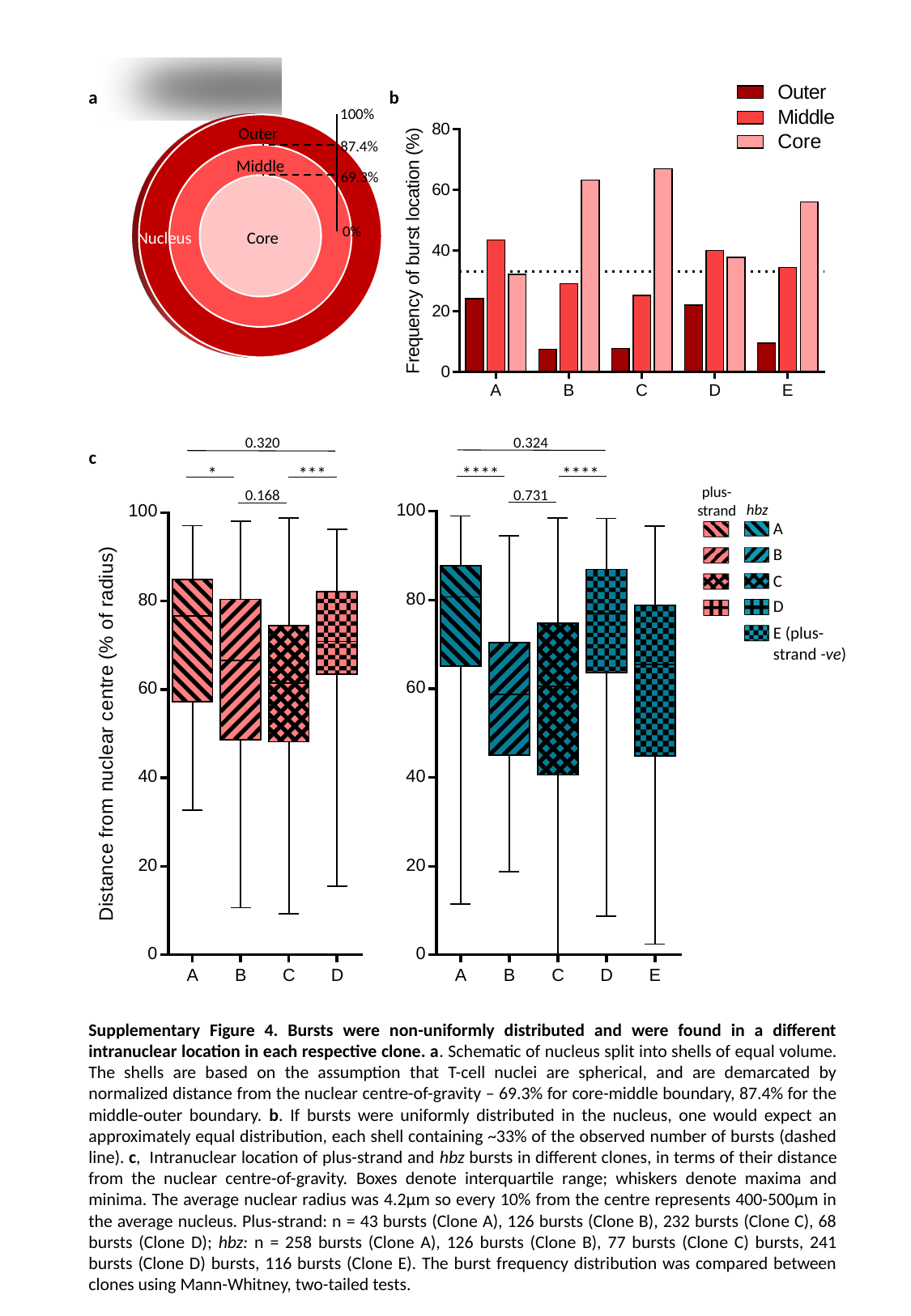

a
b
100%
Outer
87.4%
Middle
69.3%
0%
Nucleus
Core
0.320
0.324
c
*
***
****
****
plus-strand
hbz
A
B
C
D
E (plus-strand -ve)
0.168
0.731
Supplementary Figure 4. Bursts were non-uniformly distributed and were found in a different intranuclear location in each respective clone. a. Schematic of nucleus split into shells of equal volume. The shells are based on the assumption that T-cell nuclei are spherical, and are demarcated by normalized distance from the nuclear centre-of-gravity – 69.3% for core-middle boundary, 87.4% for the middle-outer boundary. b. If bursts were uniformly distributed in the nucleus, one would expect an approximately equal distribution, each shell containing ~33% of the observed number of bursts (dashed line). c, Intranuclear location of plus-strand and hbz bursts in different clones, in terms of their distance from the nuclear centre-of-gravity. Boxes denote interquartile range; whiskers denote maxima and minima. The average nuclear radius was 4.2µm so every 10% from the centre represents 400-500µm in the average nucleus. Plus-strand: n = 43 bursts (Clone A), 126 bursts (Clone B), 232 bursts (Clone C), 68 bursts (Clone D); hbz: n = 258 bursts (Clone A), 126 bursts (Clone B), 77 bursts (Clone C) bursts, 241 bursts (Clone D) bursts, 116 bursts (Clone E). The burst frequency distribution was compared between clones using Mann-Whitney, two-tailed tests.

## Slide 5
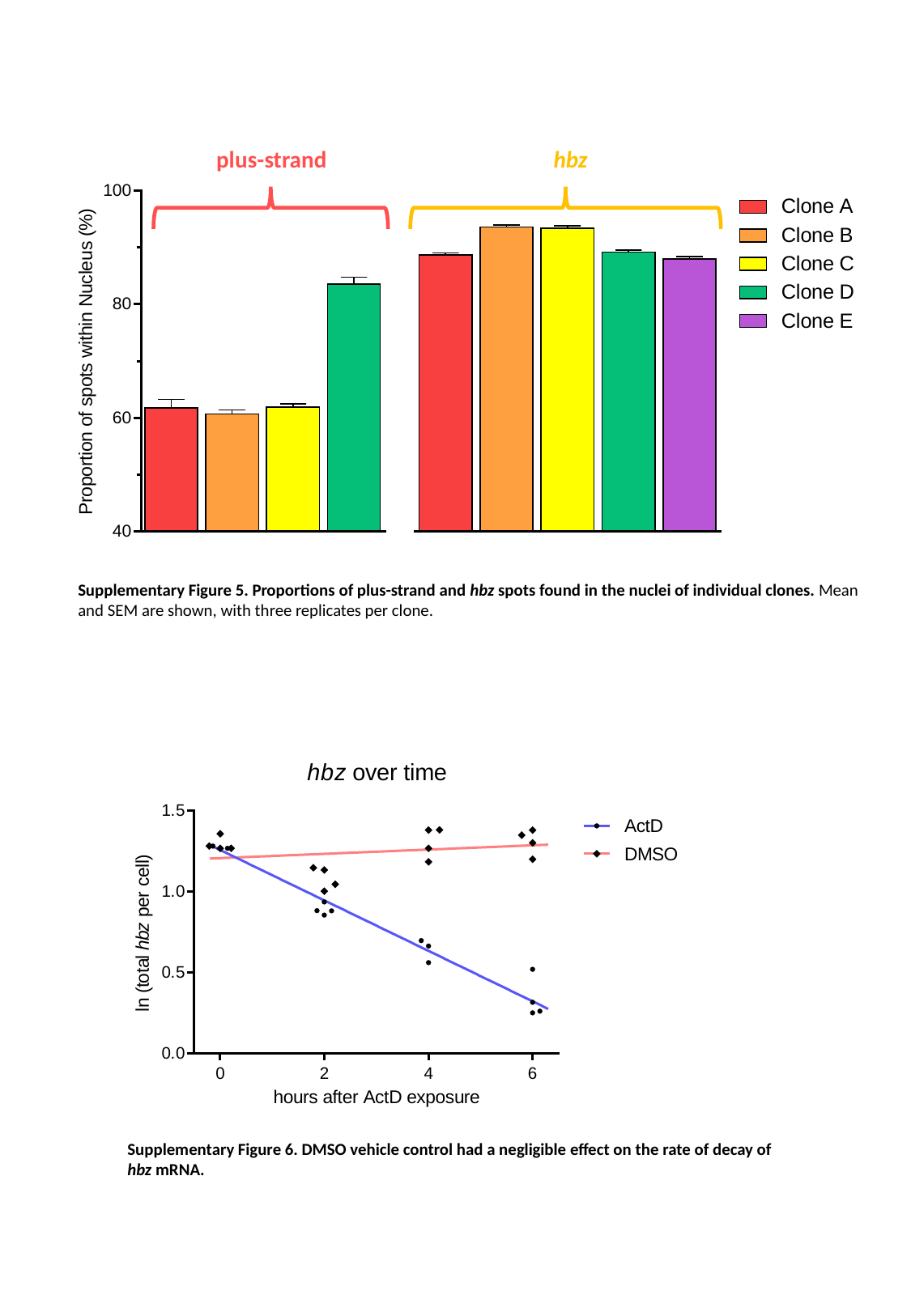

hbz
plus-strand
Supplementary Figure 5. Proportions of plus-strand and hbz spots found in the nuclei of individual clones. Mean and SEM are shown, with three replicates per clone.
Supplementary Figure 6. DMSO vehicle control had a negligible effect on the rate of decay of hbz mRNA.

## Slide 6
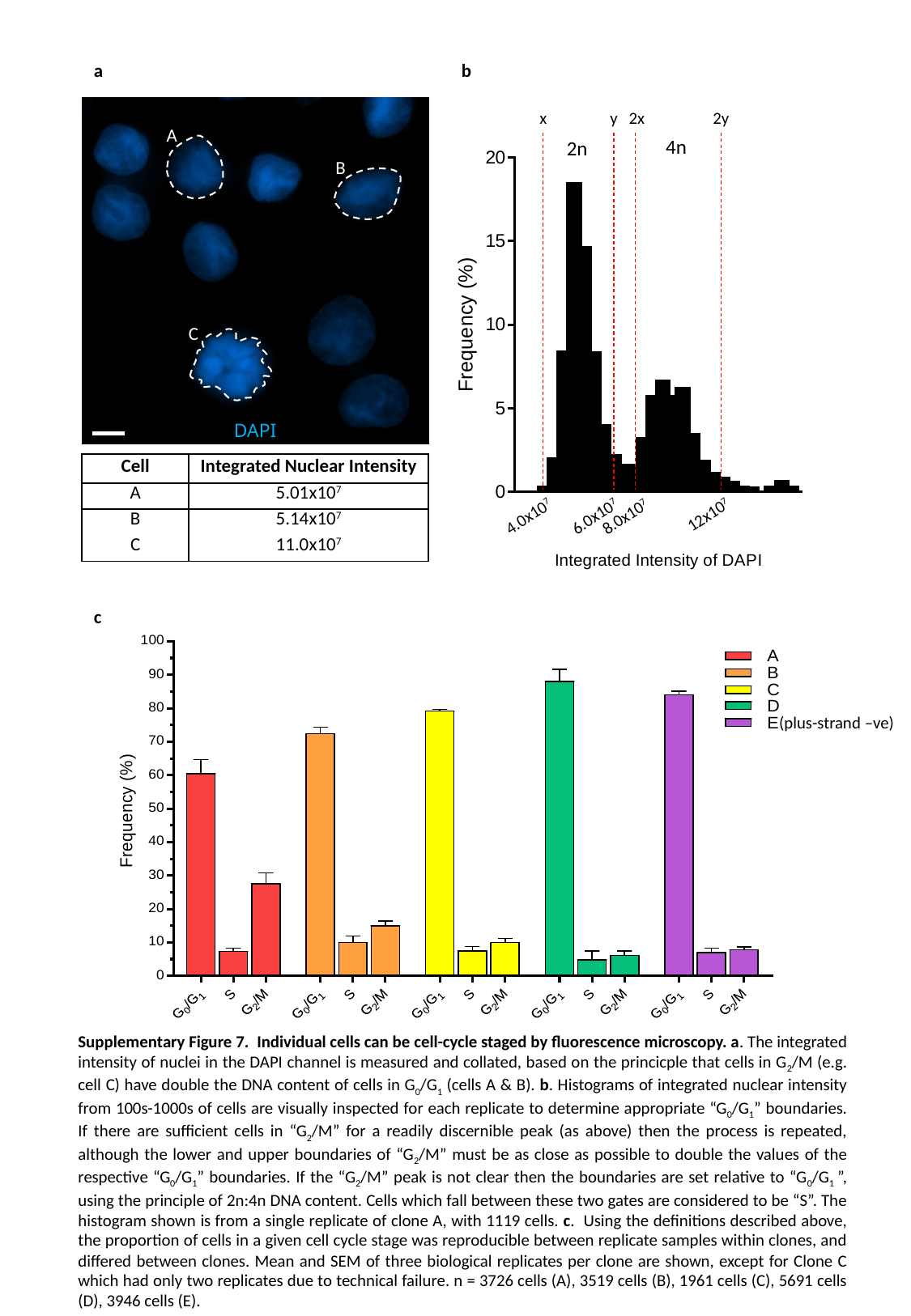

a
A
B
C
DAPI
b
x
y
2x
2y
4n
2n
12x107
4.0x10^7^
6.0x10^7^
8.0x10^7^
| Cell | Integrated Nuclear Intensity |
| --- | --- |
| A | 5.01x10^7^ |
| B | 5.14x10^7^ |
| C | 11.0x107 |
c
(plus-strand –ve)
Supplementary Figure 7. Individual cells can be cell-cycle staged by fluorescence microscopy. a. The integrated intensity of nuclei in the DAPI channel is measured and collated, based on the princicple that cells in G2/M (e.g. cell C) have double the DNA content of cells in G0/G1 (cells A & B). b. Histograms of integrated nuclear intensity from 100s-1000s of cells are visually inspected for each replicate to determine appropriate “G0/G1” boundaries. If there are sufficient cells in “G2/M” for a readily discernible peak (as above) then the process is repeated, although the lower and upper boundaries of “G2/M” must be as close as possible to double the values of the respective “G0/G1” boundaries. If the “G2/M” peak is not clear then the boundaries are set relative to “G0/G1 ”, using the principle of 2n:4n DNA content. Cells which fall between these two gates are considered to be “S”. The histogram shown is from a single replicate of clone A, with 1119 cells. c. Using the definitions described above, the proportion of cells in a given cell cycle stage was reproducible between replicate samples within clones, and differed between clones. Mean and SEM of three biological replicates per clone are shown, except for Clone C which had only two replicates due to technical failure. n = 3726 cells (A), 3519 cells (B), 1961 cells (C), 5691 cells (D), 3946 cells (E).

## Slide 7
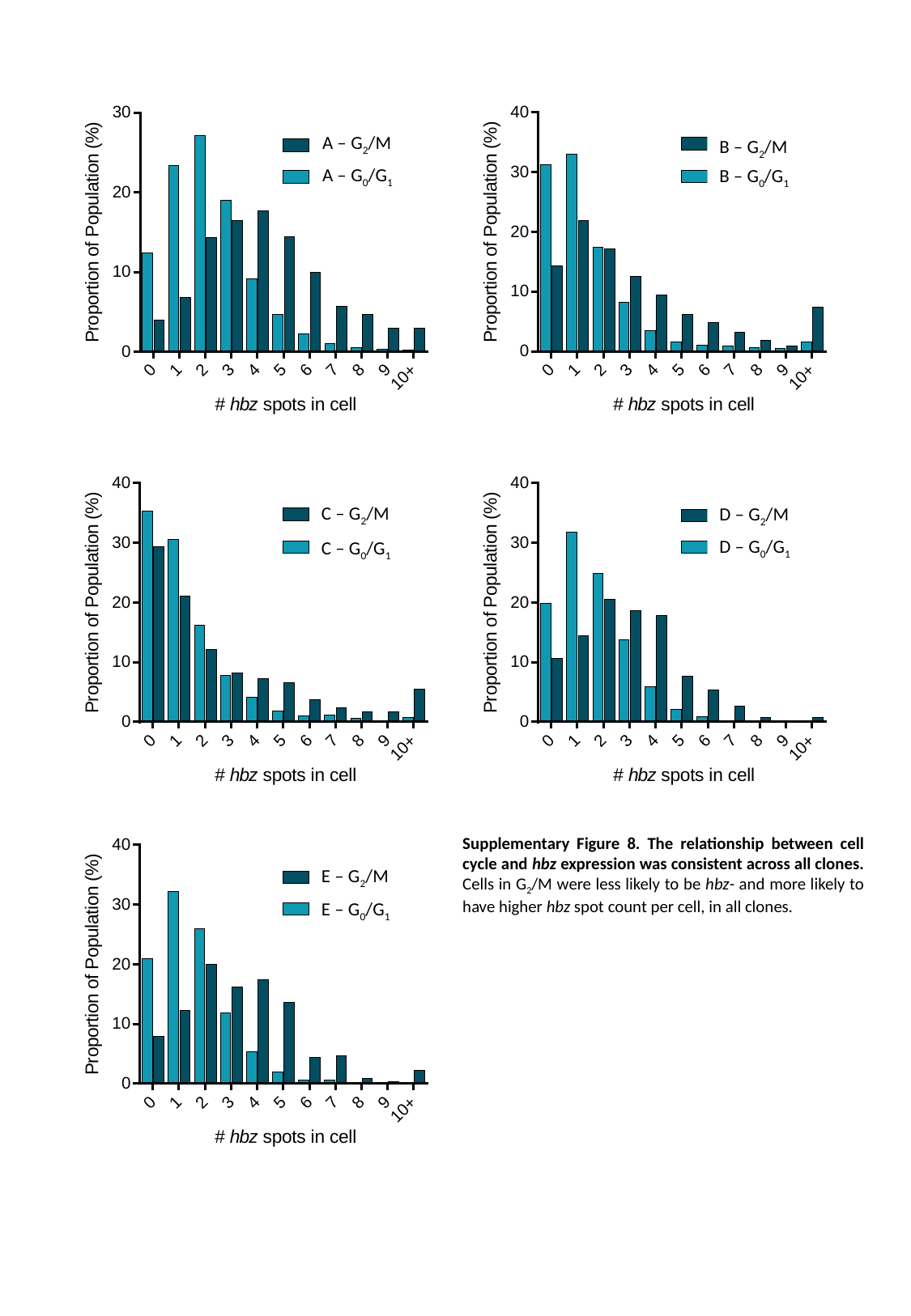

A – G2/M
B – G2/M
A – G0/G1
B – G0/G1
C – G2/M
D – G2/M
D – G0/G1
C – G0/G1
Supplementary Figure 8. The relationship between cell cycle and hbz expression was consistent across all clones. Cells in G2/M were less likely to be hbz- and more likely to have higher hbz spot count per cell, in all clones.
E – G2/M
E – G0/G1

## Slide 8
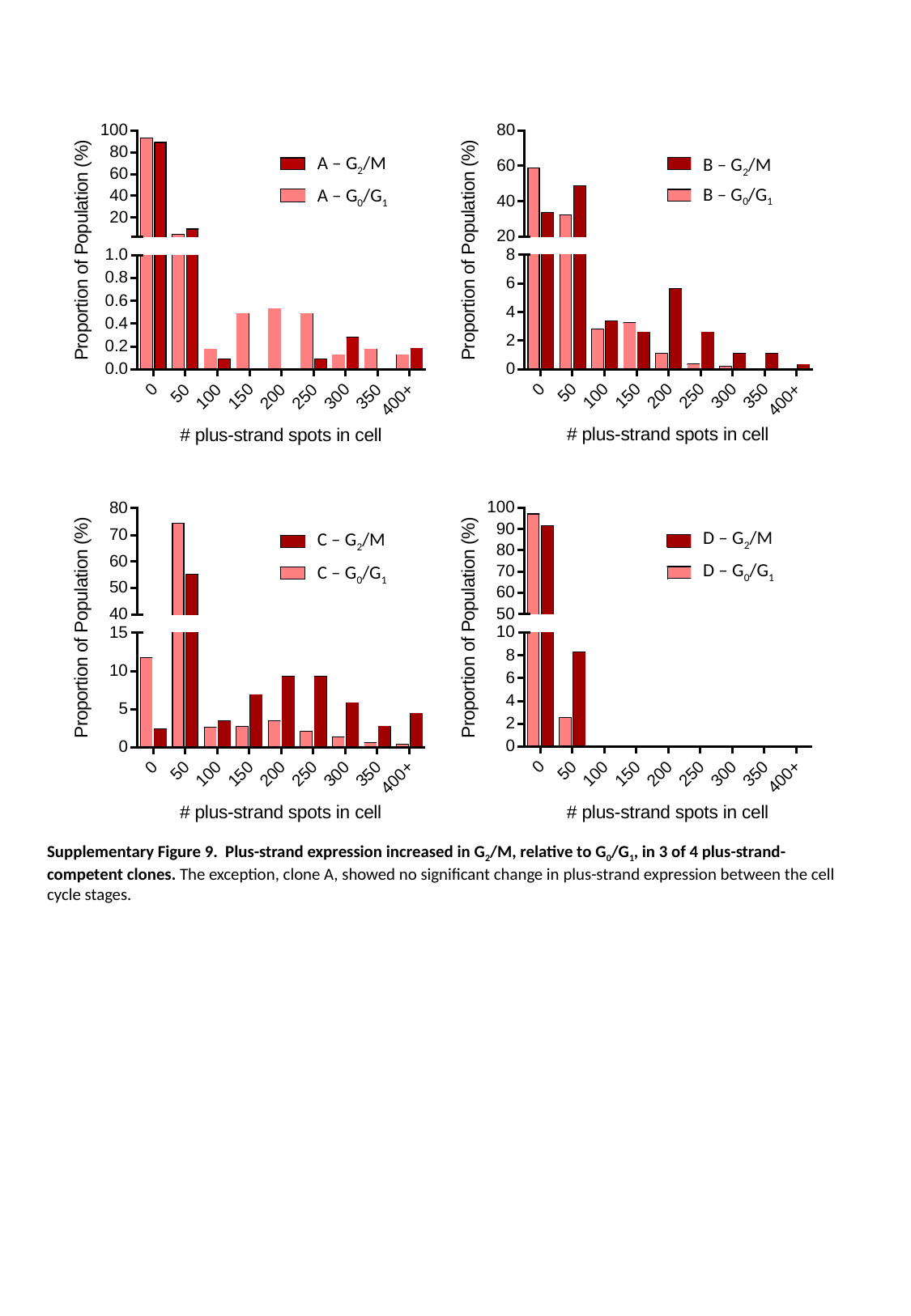

A – G2/M
B – G2/M
B – G0/G1
A – G0/G1
D – G2/M
C – G2/M
D – G0/G1
C – G0/G1
Supplementary Figure 9. Plus-strand expression increased in G2/M, relative to G0/G1, in 3 of 4 plus-strand-competent clones. The exception, clone A, showed no significant change in plus-strand expression between the cell cycle stages.

## Slide 9
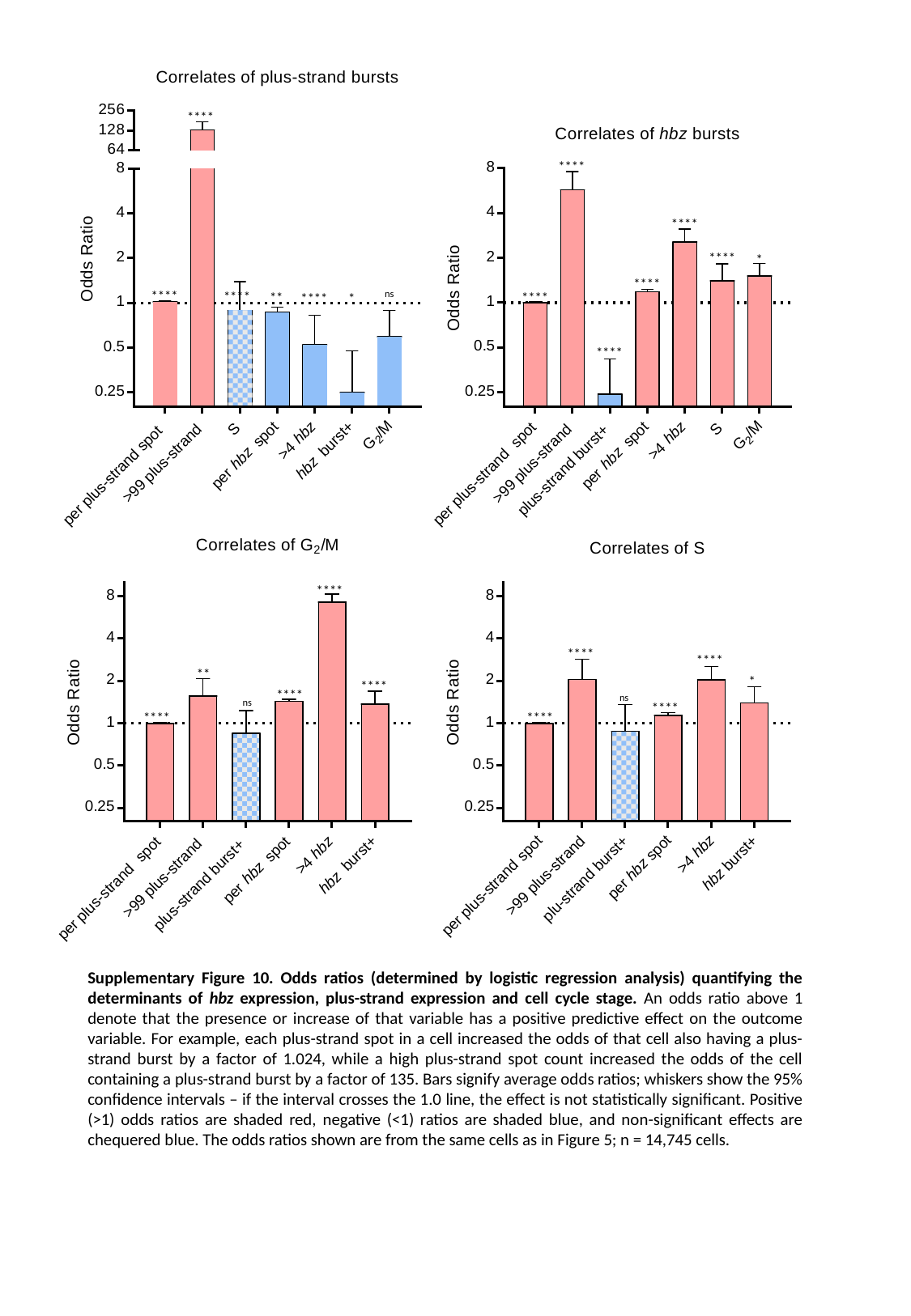

****
****
****
****
*
****
****
ns
****
****
**
****
*
****
****
****
****
**
*
****
****
ns
ns
****
****
****
Supplementary Figure 10. Odds ratios (determined by logistic regression analysis) quantifying the determinants of hbz expression, plus-strand expression and cell cycle stage. An odds ratio above 1 denote that the presence or increase of that variable has a positive predictive effect on the outcome variable. For example, each plus-strand spot in a cell increased the odds of that cell also having a plus-strand burst by a factor of 1.024, while a high plus-strand spot count increased the odds of the cell containing a plus-strand burst by a factor of 135. Bars signify average odds ratios; whiskers show the 95% confidence intervals – if the interval crosses the 1.0 line, the effect is not statistically significant. Positive (>1) odds ratios are shaded red, negative (<1) ratios are shaded blue, and non-significant effects are chequered blue. The odds ratios shown are from the same cells as in Figure 5; n = 14,745 cells.

## Slide 10
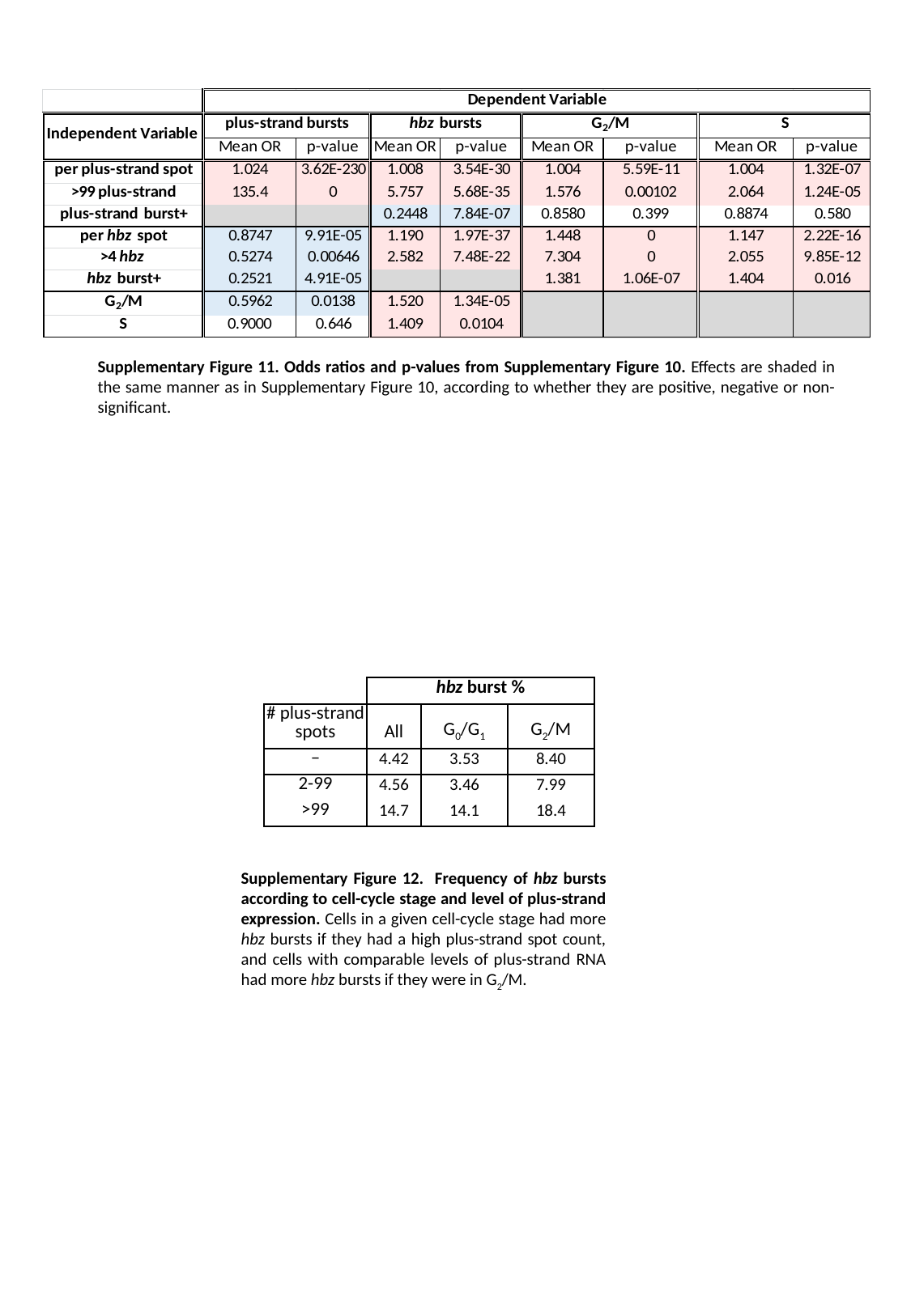

Supplementary Figure 11. Odds ratios and p-values from Supplementary Figure 10. Effects are shaded in the same manner as in Supplementary Figure 10, according to whether they are positive, negative or non-significant.
| | hbz burst % | | |
| --- | --- | --- | --- |
| # plus-strand spots | All | G0/G1 | G2/M |
| – | 4.42 | 3.53 | 8.40 |
| 2-99 | 4.56 | 3.46 | 7.99 |
| >99 | 14.7 | 14.1 | 18.4 |
Supplementary Figure 12. Frequency of hbz bursts according to cell-cycle stage and level of plus-strand expression. Cells in a given cell-cycle stage had more hbz bursts if they had a high plus-strand spot count, and cells with comparable levels of plus-strand RNA had more hbz bursts if they were in G2/M.

## Slide 11
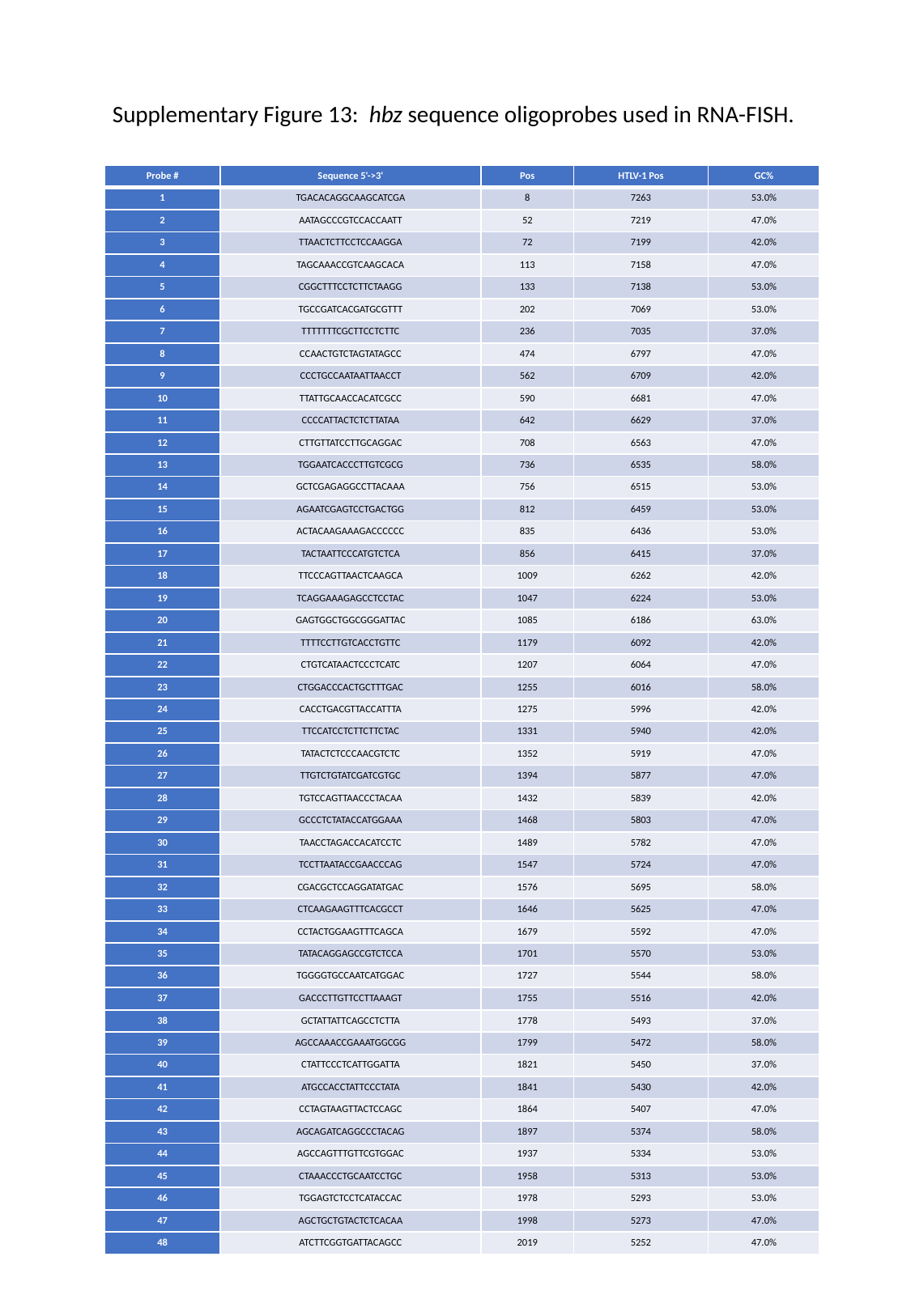

Supplementary Figure 13: hbz sequence oligoprobes used in RNA-FISH.
| Probe # | Sequence 5'->3' | Pos | HTLV-1 Pos | GC% |
| --- | --- | --- | --- | --- |
| 1 | TGACACAGGCAAGCATCGA | 8 | 7263 | 53.0% |
| 2 | AATAGCCCGTCCACCAATT | 52 | 7219 | 47.0% |
| 3 | TTAACTCTTCCTCCAAGGA | 72 | 7199 | 42.0% |
| 4 | TAGCAAACCGTCAAGCACA | 113 | 7158 | 47.0% |
| 5 | CGGCTTTCCTCTTCTAAGG | 133 | 7138 | 53.0% |
| 6 | TGCCGATCACGATGCGTTT | 202 | 7069 | 53.0% |
| 7 | TTTTTTTCGCTTCCTCTTC | 236 | 7035 | 37.0% |
| 8 | CCAACTGTCTAGTATAGCC | 474 | 6797 | 47.0% |
| 9 | CCCTGCCAATAATTAACCT | 562 | 6709 | 42.0% |
| 10 | TTATTGCAACCACATCGCC | 590 | 6681 | 47.0% |
| 11 | CCCCATTACTCTCTTATAA | 642 | 6629 | 37.0% |
| 12 | CTTGTTATCCTTGCAGGAC | 708 | 6563 | 47.0% |
| 13 | TGGAATCACCCTTGTCGCG | 736 | 6535 | 58.0% |
| 14 | GCTCGAGAGGCCTTACAAA | 756 | 6515 | 53.0% |
| 15 | AGAATCGAGTCCTGACTGG | 812 | 6459 | 53.0% |
| 16 | ACTACAAGAAAGACCCCCC | 835 | 6436 | 53.0% |
| 17 | TACTAATTCCCATGTCTCA | 856 | 6415 | 37.0% |
| 18 | TTCCCAGTTAACTCAAGCA | 1009 | 6262 | 42.0% |
| 19 | TCAGGAAAGAGCCTCCTAC | 1047 | 6224 | 53.0% |
| 20 | GAGTGGCTGGCGGGATTAC | 1085 | 6186 | 63.0% |
| 21 | TTTTCCTTGTCACCTGTTC | 1179 | 6092 | 42.0% |
| 22 | CTGTCATAACTCCCTCATC | 1207 | 6064 | 47.0% |
| 23 | CTGGACCCACTGCTTTGAC | 1255 | 6016 | 58.0% |
| 24 | CACCTGACGTTACCATTTA | 1275 | 5996 | 42.0% |
| 25 | TTCCATCCTCTTCTTCTAC | 1331 | 5940 | 42.0% |
| 26 | TATACTCTCCCAACGTCTC | 1352 | 5919 | 47.0% |
| 27 | TTGTCTGTATCGATCGTGC | 1394 | 5877 | 47.0% |
| 28 | TGTCCAGTTAACCCTACAA | 1432 | 5839 | 42.0% |
| 29 | GCCCTCTATACCATGGAAA | 1468 | 5803 | 47.0% |
| 30 | TAACCTAGACCACATCCTC | 1489 | 5782 | 47.0% |
| 31 | TCCTTAATACCGAACCCAG | 1547 | 5724 | 47.0% |
| 32 | CGACGCTCCAGGATATGAC | 1576 | 5695 | 58.0% |
| 33 | CTCAAGAAGTTTCACGCCT | 1646 | 5625 | 47.0% |
| 34 | CCTACTGGAAGTTTCAGCA | 1679 | 5592 | 47.0% |
| 35 | TATACAGGAGCCGTCTCCA | 1701 | 5570 | 53.0% |
| 36 | TGGGGTGCCAATCATGGAC | 1727 | 5544 | 58.0% |
| 37 | GACCCTTGTTCCTTAAAGT | 1755 | 5516 | 42.0% |
| 38 | GCTATTATTCAGCCTCTTA | 1778 | 5493 | 37.0% |
| 39 | AGCCAAACCGAAATGGCGG | 1799 | 5472 | 58.0% |
| 40 | CTATTCCCTCATTGGATTA | 1821 | 5450 | 37.0% |
| 41 | ATGCCACCTATTCCCTATA | 1841 | 5430 | 42.0% |
| 42 | CCTAGTAAGTTACTCCAGC | 1864 | 5407 | 47.0% |
| 43 | AGCAGATCAGGCCCTACAG | 1897 | 5374 | 58.0% |
| 44 | AGCCAGTTTGTTCGTGGAC | 1937 | 5334 | 53.0% |
| 45 | CTAAACCCTGCAATCCTGC | 1958 | 5313 | 53.0% |
| 46 | TGGAGTCTCCTCATACCAC | 1978 | 5293 | 53.0% |
| 47 | AGCTGCTGTACTCTCACAA | 1998 | 5273 | 47.0% |
| 48 | ATCTTCGGTGATTACAGCC | 2019 | 5252 | 47.0% |

## Slide 12
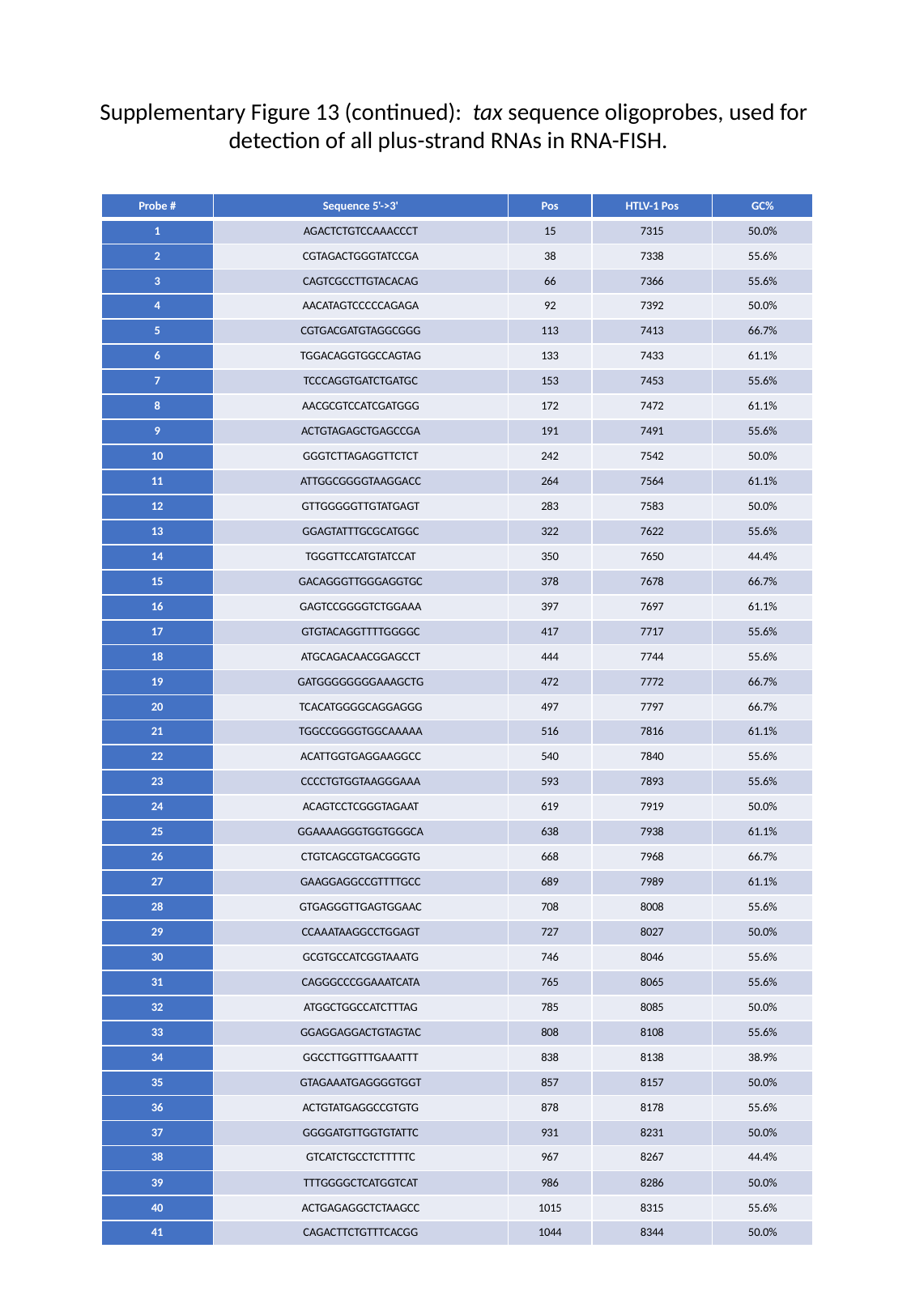

Supplementary Figure 13 (continued): tax sequence oligoprobes, used for detection of all plus-strand RNAs in RNA-FISH.
| Probe # | Sequence 5'->3' | Pos | HTLV-1 Pos | GC% |
| --- | --- | --- | --- | --- |
| 1 | AGACTCTGTCCAAACCCT | 15 | 7315 | 50.0% |
| 2 | CGTAGACTGGGTATCCGA | 38 | 7338 | 55.6% |
| 3 | CAGTCGCCTTGTACACAG | 66 | 7366 | 55.6% |
| 4 | AACATAGTCCCCCAGAGA | 92 | 7392 | 50.0% |
| 5 | CGTGACGATGTAGGCGGG | 113 | 7413 | 66.7% |
| 6 | TGGACAGGTGGCCAGTAG | 133 | 7433 | 61.1% |
| 7 | TCCCAGGTGATCTGATGC | 153 | 7453 | 55.6% |
| 8 | AACGCGTCCATCGATGGG | 172 | 7472 | 61.1% |
| 9 | ACTGTAGAGCTGAGCCGA | 191 | 7491 | 55.6% |
| 10 | GGGTCTTAGAGGTTCTCT | 242 | 7542 | 50.0% |
| 11 | ATTGGCGGGGTAAGGACC | 264 | 7564 | 61.1% |
| 12 | GTTGGGGGTTGTATGAGT | 283 | 7583 | 50.0% |
| 13 | GGAGTATTTGCGCATGGC | 322 | 7622 | 55.6% |
| 14 | TGGGTTCCATGTATCCAT | 350 | 7650 | 44.4% |
| 15 | GACAGGGTTGGGAGGTGC | 378 | 7678 | 66.7% |
| 16 | GAGTCCGGGGTCTGGAAA | 397 | 7697 | 61.1% |
| 17 | GTGTACAGGTTTTGGGGC | 417 | 7717 | 55.6% |
| 18 | ATGCAGACAACGGAGCCT | 444 | 7744 | 55.6% |
| 19 | GATGGGGGGGGAAAGCTG | 472 | 7772 | 66.7% |
| 20 | TCACATGGGGCAGGAGGG | 497 | 7797 | 66.7% |
| 21 | TGGCCGGGGTGGCAAAAA | 516 | 7816 | 61.1% |
| 22 | ACATTGGTGAGGAAGGCC | 540 | 7840 | 55.6% |
| 23 | CCCCTGTGGTAAGGGAAA | 593 | 7893 | 55.6% |
| 24 | ACAGTCCTCGGGTAGAAT | 619 | 7919 | 50.0% |
| 25 | GGAAAAGGGTGGTGGGCA | 638 | 7938 | 61.1% |
| 26 | CTGTCAGCGTGACGGGTG | 668 | 7968 | 66.7% |
| 27 | GAAGGAGGCCGTTTTGCC | 689 | 7989 | 61.1% |
| 28 | GTGAGGGTTGAGTGGAAC | 708 | 8008 | 55.6% |
| 29 | CCAAATAAGGCCTGGAGT | 727 | 8027 | 50.0% |
| 30 | GCGTGCCATCGGTAAATG | 746 | 8046 | 55.6% |
| 31 | CAGGGCCCGGAAATCATA | 765 | 8065 | 55.6% |
| 32 | ATGGCTGGCCATCTTTAG | 785 | 8085 | 50.0% |
| 33 | GGAGGAGGACTGTAGTAC | 808 | 8108 | 55.6% |
| 34 | GGCCTTGGTTTGAAATTT | 838 | 8138 | 38.9% |
| 35 | GTAGAAATGAGGGGTGGT | 857 | 8157 | 50.0% |
| 36 | ACTGTATGAGGCCGTGTG | 878 | 8178 | 55.6% |
| 37 | GGGGATGTTGGTGTATTC | 931 | 8231 | 50.0% |
| 38 | GTCATCTGCCTCTTTTTC | 967 | 8267 | 44.4% |
| 39 | TTTGGGGCTCATGGTCAT | 986 | 8286 | 50.0% |
| 40 | ACTGAGAGGCTCTAAGCC | 1015 | 8315 | 55.6% |
| 41 | CAGACTTCTGTTTCACGG | 1044 | 8344 | 50.0% |

## Slide 13
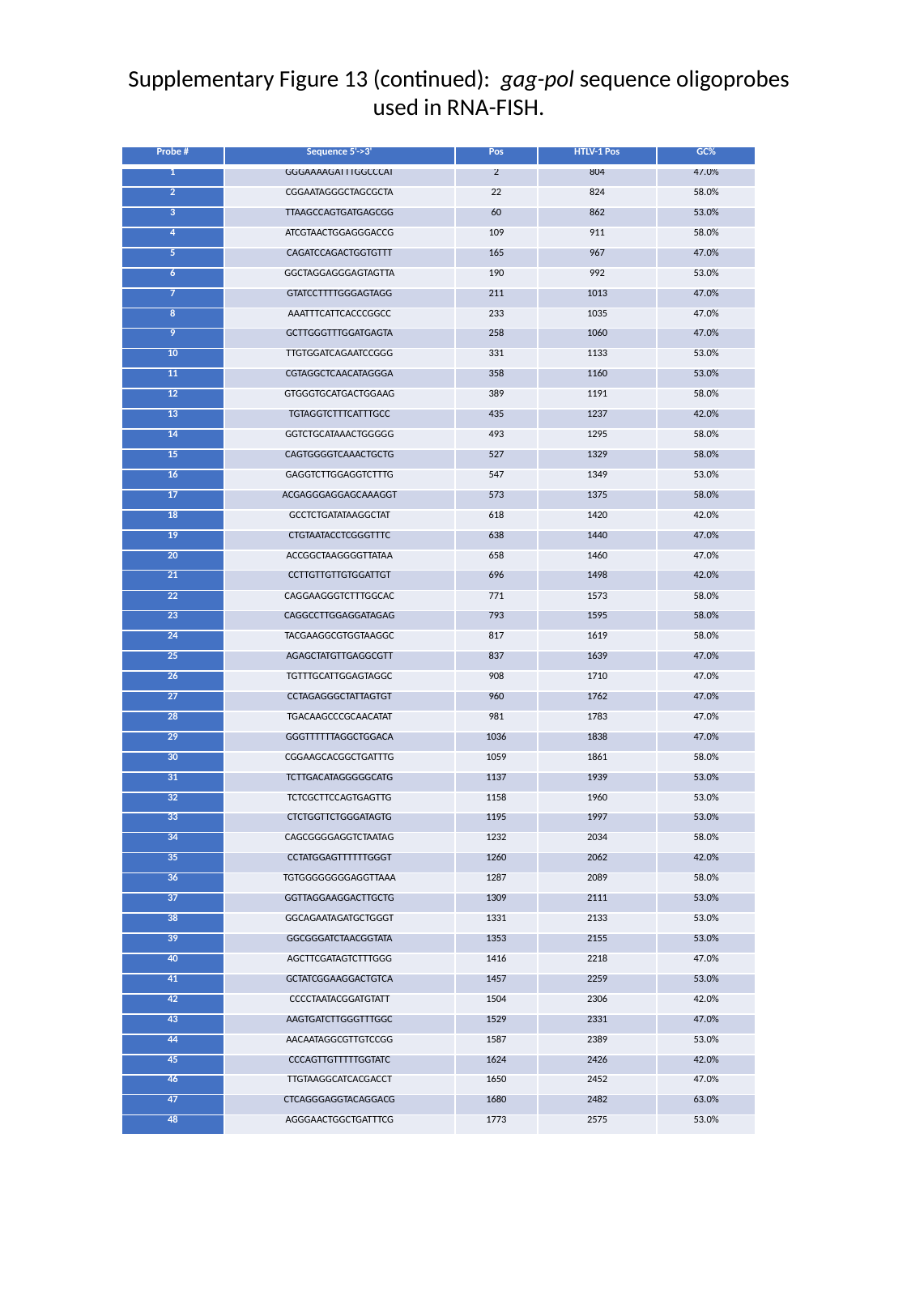

Supplementary Figure 13 (continued): gag-pol sequence oligoprobes used in RNA-FISH.
| Probe # | Sequence 5'->3' | Pos | HTLV-1 Pos | GC% |
| --- | --- | --- | --- | --- |
| 1 | GGGAAAAGATTTGGCCCAT | 2 | 804 | 47.0% |
| 2 | CGGAATAGGGCTAGCGCTA | 22 | 824 | 58.0% |
| 3 | TTAAGCCAGTGATGAGCGG | 60 | 862 | 53.0% |
| 4 | ATCGTAACTGGAGGGACCG | 109 | 911 | 58.0% |
| 5 | CAGATCCAGACTGGTGTTT | 165 | 967 | 47.0% |
| 6 | GGCTAGGAGGGAGTAGTTA | 190 | 992 | 53.0% |
| 7 | GTATCCTTTTGGGAGTAGG | 211 | 1013 | 47.0% |
| 8 | AAATTTCATTCACCCGGCC | 233 | 1035 | 47.0% |
| 9 | GCTTGGGTTTGGATGAGTA | 258 | 1060 | 47.0% |
| 10 | TTGTGGATCAGAATCCGGG | 331 | 1133 | 53.0% |
| 11 | CGTAGGCTCAACATAGGGA | 358 | 1160 | 53.0% |
| 12 | GTGGGTGCATGACTGGAAG | 389 | 1191 | 58.0% |
| 13 | TGTAGGTCTTTCATTTGCC | 435 | 1237 | 42.0% |
| 14 | GGTCTGCATAAACTGGGGG | 493 | 1295 | 58.0% |
| 15 | CAGTGGGGTCAAACTGCTG | 527 | 1329 | 58.0% |
| 16 | GAGGTCTTGGAGGTCTTTG | 547 | 1349 | 53.0% |
| 17 | ACGAGGGAGGAGCAAAGGT | 573 | 1375 | 58.0% |
| 18 | GCCTCTGATATAAGGCTAT | 618 | 1420 | 42.0% |
| 19 | CTGTAATACCTCGGGTTTC | 638 | 1440 | 47.0% |
| 20 | ACCGGCTAAGGGGTTATAA | 658 | 1460 | 47.0% |
| 21 | CCTTGTTGTTGTGGATTGT | 696 | 1498 | 42.0% |
| 22 | CAGGAAGGGTCTTTGGCAC | 771 | 1573 | 58.0% |
| 23 | CAGGCCTTGGAGGATAGAG | 793 | 1595 | 58.0% |
| 24 | TACGAAGGCGTGGTAAGGC | 817 | 1619 | 58.0% |
| 25 | AGAGCTATGTTGAGGCGTT | 837 | 1639 | 47.0% |
| 26 | TGTTTGCATTGGAGTAGGC | 908 | 1710 | 47.0% |
| 27 | CCTAGAGGGCTATTAGTGT | 960 | 1762 | 47.0% |
| 28 | TGACAAGCCCGCAACATAT | 981 | 1783 | 47.0% |
| 29 | GGGTTTTTTAGGCTGGACA | 1036 | 1838 | 47.0% |
| 30 | CGGAAGCACGGCTGATTTG | 1059 | 1861 | 58.0% |
| 31 | TCTTGACATAGGGGGCATG | 1137 | 1939 | 53.0% |
| 32 | TCTCGCTTCCAGTGAGTTG | 1158 | 1960 | 53.0% |
| 33 | CTCTGGTTCTGGGATAGTG | 1195 | 1997 | 53.0% |
| 34 | CAGCGGGGAGGTCTAATAG | 1232 | 2034 | 58.0% |
| 35 | CCTATGGAGTTTTTTGGGT | 1260 | 2062 | 42.0% |
| 36 | TGTGGGGGGGGAGGTTAAA | 1287 | 2089 | 58.0% |
| 37 | GGTTAGGAAGGACTTGCTG | 1309 | 2111 | 53.0% |
| 38 | GGCAGAATAGATGCTGGGT | 1331 | 2133 | 53.0% |
| 39 | GGCGGGATCTAACGGTATA | 1353 | 2155 | 53.0% |
| 40 | AGCTTCGATAGTCTTTGGG | 1416 | 2218 | 47.0% |
| 41 | GCTATCGGAAGGACTGTCA | 1457 | 2259 | 53.0% |
| 42 | CCCCTAATACGGATGTATT | 1504 | 2306 | 42.0% |
| 43 | AAGTGATCTTGGGTTTGGC | 1529 | 2331 | 47.0% |
| 44 | AACAATAGGCGTTGTCCGG | 1587 | 2389 | 53.0% |
| 45 | CCCAGTTGTTTTTGGTATC | 1624 | 2426 | 42.0% |
| 46 | TTGTAAGGCATCACGACCT | 1650 | 2452 | 47.0% |
| 47 | CTCAGGGAGGTACAGGACG | 1680 | 2482 | 63.0% |
| 48 | AGGGAACTGGCTGATTTCG | 1773 | 2575 | 53.0% |

## Slide 14
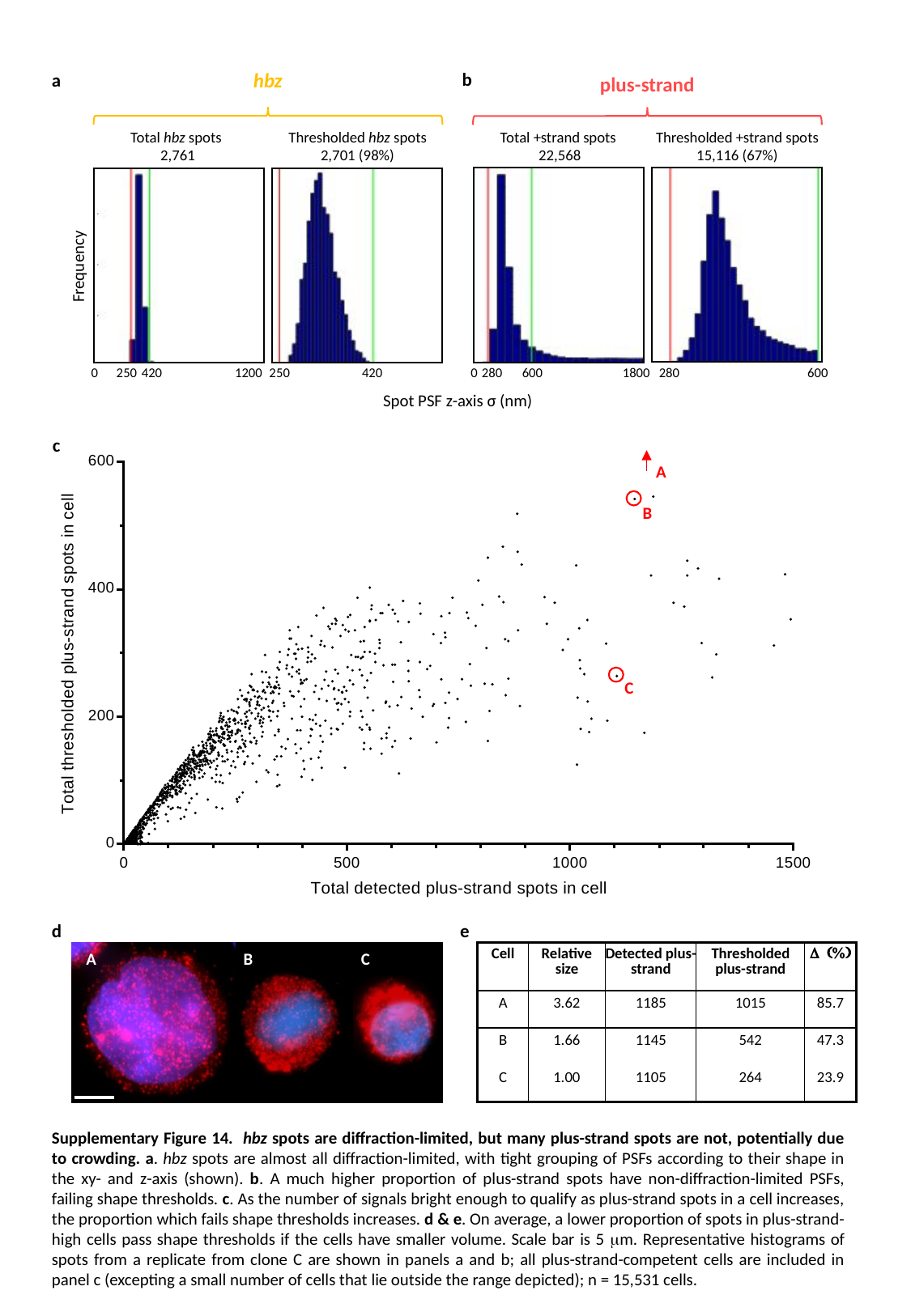

hbz
plus-strand
Total hbz spots
2,761
Thresholded hbz spots 2,701 (98%)
Total +strand spots
 22,568
Thresholded +strand spots 15,116 (67%)
600
280
250
420
Frequency
0
250
420
1200
0
280
600
1800
Spot PSF z-axis σ (nm)
b
a
c
A
B
C
d
e
A
B
C
| Cell | Relative size | Detected plus-strand | Thresholded plus-strand | D (%) |
| --- | --- | --- | --- | --- |
| A | 3.62 | 1185 | 1015 | 85.7 |
| B | 1.66 | 1145 | 542 | 47.3 |
| C | 1.00 | 1105 | 264 | 23.9 |
Supplementary Figure 14. hbz spots are diffraction-limited, but many plus-strand spots are not, potentially due to crowding. a. hbz spots are almost all diffraction-limited, with tight grouping of PSFs according to their shape in the xy- and z-axis (shown). b. A much higher proportion of plus-strand spots have non-diffraction-limited PSFs, failing shape thresholds. c. As the number of signals bright enough to qualify as plus-strand spots in a cell increases, the proportion which fails shape thresholds increases. d & e. On average, a lower proportion of spots in plus-strand-high cells pass shape thresholds if the cells have smaller volume. Scale bar is 5 mm. Representative histograms of spots from a replicate from clone C are shown in panels a and b; all plus-strand-competent cells are included in panel c (excepting a small number of cells that lie outside the range depicted); n = 15,531 cells.

## Slide 15
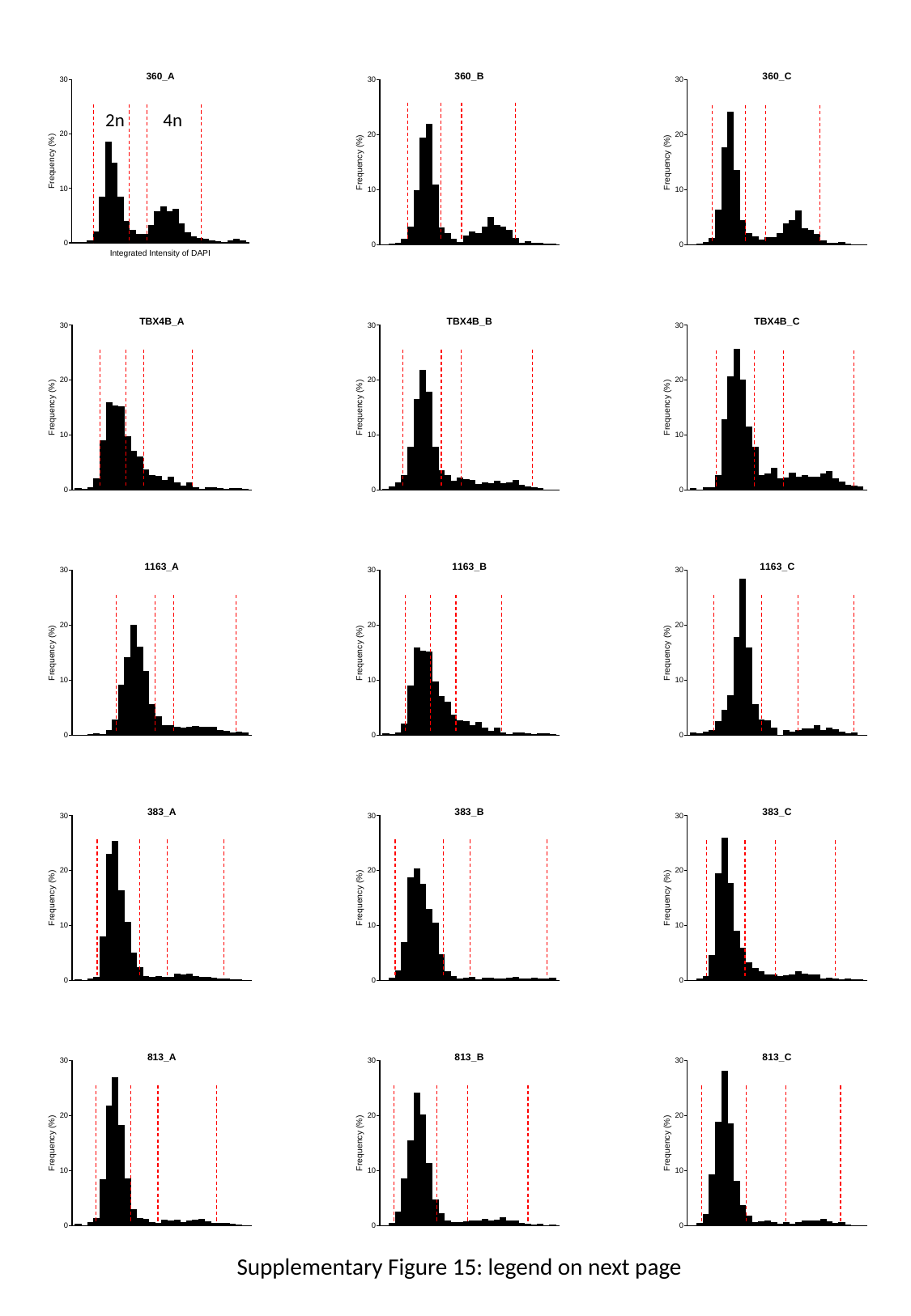

2n
4n
Supplementary Figure 15: legend on next page

## Slide 16
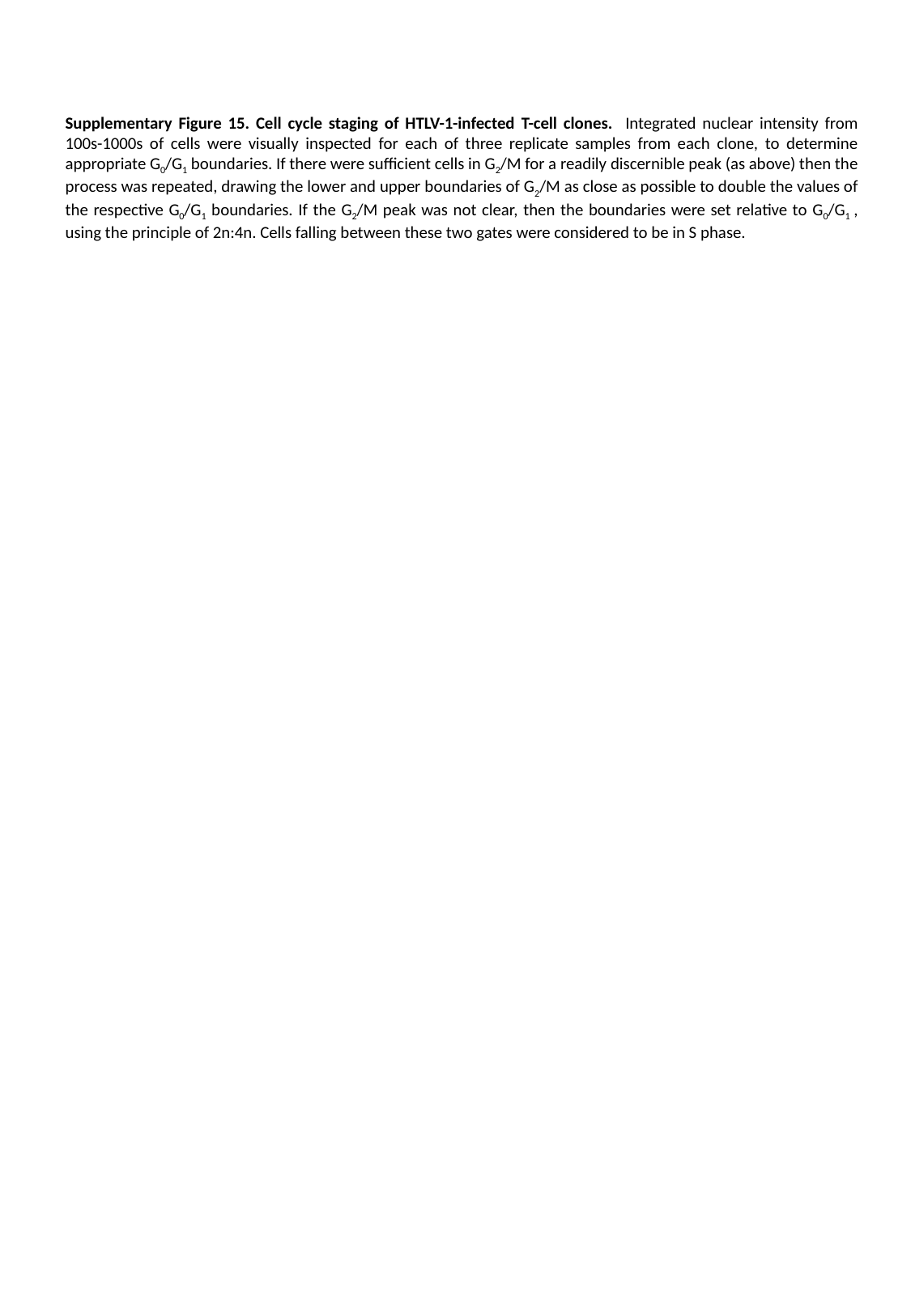

Supplementary Figure 15. Cell cycle staging of HTLV-1-infected T-cell clones. Integrated nuclear intensity from 100s-1000s of cells were visually inspected for each of three replicate samples from each clone, to determine appropriate G0/G1 boundaries. If there were sufficient cells in G2/M for a readily discernible peak (as above) then the process was repeated, drawing the lower and upper boundaries of G2/M as close as possible to double the values of the respective G0/G1 boundaries. If the G2/M peak was not clear, then the boundaries were set relative to G0/G1 , using the principle of 2n:4n. Cells falling between these two gates were considered to be in S phase.
