# Supplementary material for: Single-cell heterogeneity and cell-cycle-related viral gene bursts in the human leukaemia virus HTLV-1
Source: Wellcome Open Res. 2017 Dec 11;2:87. Originally published 2017 Sep 22. [Version 2] doi: 10.12688/wellcomeopenres.12469.2 (PMC5645716; doi:10.12688/wellcomeopenres.12469.2)
Supplement: Peer review history

# Peer review: Referee response for version 1

**DOI:** 10.21956/wellcomeopenres.13502.r26509

**Published:** 2017-11-01

**Authors:** Marc Sitbon

**Affiliations:** 1 UMR 5535, CNRS, Institute of Molecular Genetics of Montpellier, Montpellier, France

Billman *et al.* report here a detailed analysis of plus-strand (+strand) " *tax*" and anti-sense ( *hbz*) RNA transcripts in HTLV-1 T cell clones that have been cultured *ex vivo* from CD4+CD25+ T cells isolated from PBMCs of 4 different patients. Each clone, harboring a single entire copy of the HTLV-1 genome, was cultured under periodic activation with IL-2, and anti CD2, CD3 and CD28 mAbs. All analyses were performed at day 8 of the 14 day activation cycle, when most cells are returning to a resting state. Single cell analyses of HTLV-1 RNA expression were based on integration of single molecule RNA-FISH and DAPI nuclear staining with occasional actinomycin D treatment.

 Under these conditions, the authors observed that the vast majority of cells harbored detectable levels of *hbz *transcripts, while accumulation of +strand transcripts (which they refer to as " *tax*", see below) was more heterogeneous, varying from <10% in cells from 2 of the clones, to 50-60% of cells from the other 2 clones. An additional clone that harbored a deleted provirus, and thereby did not produce +strand transcripts, served as control. The authors further showed that both sense and anti-sense transcripts could be detected within larger nuclear spots in 4 to 7% of the cells analyzed, which the authors interpreted as transcriptional bursts; this interpretation was confirmed by the significant drop of bursts observed after treatment with actinomycin D. Act D further allowed the authors to show that +strand " *tax*" transcripts were rapidly accumulating to the cytoplasm while *hbz *transcripts remained in the nucleus. According to the authors, and in agreement with former observations by several teams, *hbz* and *tax* transcripts were in general inversely related. Also, the authors report that both types of transcripts tended to accumulate in cells in G2/M as compared to G0/G1 (monitored as a function of DAPI signal).

 Elucidation of the *in vivo* dynamics of Hbz versus Tax production at the single cell level should indeed help elucidate the *in vivo* regulation of latent versus productive HTLV-1 infection, and provide precious clues on the pathogenic outcomes following infection. Despite an impressive amount of data cross-analyses based on +strand and anti-sense transcription of T cell clones derived from patients' PBMCs, the actual relevance of the model for *in vivo* situations is questionable. Thus, all analyses were performed on 4 clones isolated from a subpopulation of CD4+CD25+ T cells (as described in a reference and not mentioned here), which after being isolated from patients PBMCs, were cultured for a long period of time in conditions that do not reproduce *in vivo* conditions (hyperoxia, hyperglycemia, strong recurrent activations and absence of immune pressure). As such, several of the authors' conclusions (in the title, abstract and discussion) are not warranted (see below).

 As presented, the study brings up other several additional points:

1. The authors state that all HTLV-1 +strand transcripts are detected with the so-called " *tax*" probe. Nevertheless, the constant designation of the transcripts as "tax" throughout the article can be very misleading. It would be more accurate to refer to the results obtained with this probe in the text and figures as "+strand", or another more inclusive designation, rather than " *tax*" solely.
2. Nearly 60 to 90% of cells in all 5 clones express *hbz *antisense transcripts, but depending on the clone studied, the presence of +strand transcripts, as detected with the " *tax" *probe, are much more heterogeneous (<10% in clones A and D, and 50-60% in clones B & C). Given this overwhelming heterogeneity, how can the authors exclude that it is not due to variation in Tax-encoding transcripts, but solely to other viral +strand transcripts. In other words, the authors cannot exclude that levels of Tax-encoding transcripts were homogeneously low in all clones.
3. Although the authors insist on the pitfalls of qPCR to analyze single cell transcription, there have been several reports on microfluidics technology being successfully used to monitor single cell RNA expression (i.e. Moussy *et al*. 2017. PLoS  Biol 15(7):e2001867). The application of microfluidics on PBMCs directly isolated from patients would likely allow a more accurate assessment of the *in vivo* status of RNA transcripts.
4. As mentioned above, given the experimental model of subcloning and culture (at the non-physiological 20% oxygen tension, which artificially modulates HTLV-1 activation, as previously published by the authors), and in the absence of immune pressure, the claim that the results presented here are relevant for *in vivo* situations and " *offer an explanation for the paradoxical correlations observed between the host immune response and HTLV-1 transcription*" is not warranted.
5. Since the correlation between "transcription bursts" and the G2/M cell cycle stage is not established for +strand "tax" transcripts, and pertains to <7% of cells in which *hbz* is detected, the claim of a relationship between viral gene bursts and cell cycle (as stated in the general conclusion and the title) does not appear justified. Therefore, the intricate array of cross analyses and statistical evaluation performed here and addressed lengthily in the discussion is not likely to reflect *in vivo* situations.
6. Statistical significance of the correlation between high *hbz *expression, *hbz* bursts and the G2/M stages should be directly mentioned in Fig. 5 and the corresponding text. The clones used to obtain the data provided in panel 5b are not indicated (it appears that clones A and B were used here, whereas data in 5c are mentioned in the figure legend to result from the average of clones A, B, C and D).
7. Given the reservations mentioned above (an *in vitro* system that does not recapitulate *in vivo *conditions, the lack of distinction between *tax *versus +strand transcripts, and the lack of data on Tat and HBZ proteins), the discussion remains too speculative.
8. In this study, there is no assessment of the relationship between transcript and protein levels; moreover, since the half-lives of Tax and Hbz proteins in this model are not established, it is not possible to evaluate the actual timing of a potential cross-regulation between the 2 proteins. In this regard, the authors should discuss a recent paper by Baratella *et al.* (PLos Negl Trop Dis Jan 2017) that describes the specific accumulation of the HBZ protein in the cytoplasm of PBMCs directly isolated from TSP/HAM patients.
9. In suppl. fig 7 - there is an SEM indicated in the histogram of clone C while there was only 2 replicates, according to the figure legend. Also, in suppl. Figs 8 and 9 there appears to be a color and legend inversion for clones B, C, and D, and clone A does not seem to fit the G2/M correlation with high levels of +strand "tax" transcripts.

I have read this submission. I believe that I have an appropriate level of expertise to confirm that it is of an acceptable scientific standard, however I have significant reservations, as outlined above.

# Peer review: Referee response for version 1

**DOI:** 10.21956/wellcomeopenres.13502.r26294

**Published:** 2017-10-16

**Authors:** Ali Bazarbachi, Hiba El Hajj

**Affiliations:** 1 Department of Internal Medicine, American University of Beirut, Beirut, Lebanon

This is a well-written report from leading investigators in the field of HTLV-I replication. In this study, the authors screened more than 19 000 cells derived from five naturally infected T cell clones isolated from PBMCs of HTLV-1 healthy donors. They used single-molecule RNA FISH to quantify, at the single cell level, the transcripts of two main products of the HTLV-I pX region, Tax encoded by the plus-strand of the provirus and HBZ encoded by its minus-strand. They observed a strong intra- and inter-clonal heterogeneity in the expression of *tax* and *hbz *genes with Tax being expressed at high levels in few cells whereas *hbz* is expressed at lower levels in most cells. They also report that both genes are transcribed in intermittent bursts and that tax expression is enhanced in the absence of HBZ but that *hbz* expression is enhanced by Tax. Finally, they show that HBZ expression is mostly associated with G2/M phases of the cell cycle, and that its abundance correlates with the nuclear volume. They conclude that the main function of this frequent low level of *hbz* transcript and protein is to maintain clonal persistence and that conversely, intense transient intermittent bursts of *tax* drive viral replication and clonal proliferation while escaping the robust anti Tax CTL response. These results strongly suggest that the plus strand of HTLV-I is expressed in bursts *in vivo* explaining the hitherto unexplained paradoxical observations of a strong anti-Tax immune response in all immuno-competent HTLV-I infected individuals while Tax is undetectable in their freshly isolated PBMCs. This is an important study in the field that defeats multiple previous paradigms. The following experiments should strengthen the manuscript:

  

1. Authors used short-term cultures of activated cells in order to isolate individual cells. However, they need to show *tax* and *hbz* expression at the single cell level in freshly isolated PBMCs since cell culture and activation are likely to affect *tax* and *hbz *expression.
2. Figure 1: It is surprising that 1% of uninfected clones (negative control) express 50 *tax *transcripts and only 1 *hbz* transcript. More uninfected clones should be tested. Furthermore, in order to validate *tax* and *hbz *probes, they show the specificity/sensitivity of smFISH on one clone generated from HTLV-I infected cell lines like having different expression levels of Tax and HBZ.
3. It is important to show the viral load and the status of expression of Tax and HBZ in total PBMCs of the 5 HTLV-I positive donors, and compare it with that of the 5 T cell clones that were selected. How can the authors be sure that the selected clone from each donor is a representative one of all the HTLV-1 infected cells in that individual? Did they screen other clones from each of the five donors?
4. Figure 2: Images of *tax* bursts are more convincing that those of *hbz*. Figure 2D shows that the estimated number of *hbz* RNA per burst is around 3 to 4 with little intercellular variation, which is not classical for bursts. How can the authors rule out the possibility that these represent superposition of multiple transcripts. In 2C, they show a three-fold reduction in the frequency of *hbz* bursts in the presence of actinomycine D compared to a total disappearance of *tax* bursts. What is the percentage reduction of *hbz* bursts relative to the reduction of total *hbz *transcripts?
5. Figure 3: Estimation of intranuclear mRNA should be done in zStacks to rule out counting transcripts above and below the nucleus.
6. Figure 4: Authors show increased frequency of *hbz *bursts in high tax positive cells. Again, what about the total amount of *hbz* transcripts in these cells?
7. Elevated *tax *and *hbz *expression in S and G2/M phase of the cell cycle is solely based on the integrated intensity of the DAPI stained nucleus (2n versus 4n). It would be nice to perform synchronization of cells to confirm these findings.
8. Supplementary Figure 9: The negative control (uninfected cells) and clone E should be shown.
9. Supplementary Figure 10: The odds ratio for G2/M phase is much higher for cells with >4 *hbz *compared to cells with >99 tax whereas that for S phase is quite similar. Does this indicate that *hbz* expression results in G2/M arrest? Is there any correlation between G2/M phase and nuclear size in these cells?

We have read this submission. We believe that we have an appropriate level of expertise to confirm that it is of an acceptable scientific standard.

# Peer review: Referee response for version 1

**DOI:** 10.21956/wellcomeopenres.13502.r26508

**Published:** 2017-10-12

**Authors:** Vincenzo Ciminale

**Affiliations:** 1 Department of Surgery, Oncology and Gastroenterology, University of Padua, Padua, Italy

In the present study Billman *et al.* used single-molecule RNA-FISH to quantify the expression of HTLV-1 transcripts at the single-cell level. This analysis was carried out in five T-cell clones isolated by limiting dilution from peripheral blood of HTLV-1-infected subjects. Results revealed strong heterogeneity in plus- and minus- strand expression of the viral genome both within and between clones, suggesting that viral genes are transcribed in transcriptional bursts. Interestingly, *hbz *expression was associated with the S and G /M phases of the cell cycle, and was correlated with nuclear volume.

 In general, this is an excellent study that applies state-of the-art techniques to tackle some key unanswered questions on the regulation of HTLV-1 gene expression and their possible connection with the viral persistence strategies and pathogenic properties *in vivo*.

 The study is very thorough and the data presented are solid and convincing. I have a few suggestions that the Authors might want to consider to improve the quality of this paper.

1. It would be useful to indicate of the location of the probes on the scheme of the genome shown in Fig. 1A.
2. Most of the Tax probes will hybridize to ALL plus-strand transcripts, including the highly expressed *gag*/ *pol*, *env* and p21 mRNAs. Although the authors clearly state that “tax is used as a proxy for plus-strand proviral expression” referring to *Tax* may be misleading and is an oversimplification that does not fit well such a careful and thorough study. Indicating this signal as “plus-strand” throughout the paper would be more appropriate.
3. Immunofluorescence images should also show the individual channels, not just the overlay.
4. Although the association between a single mRNA molecule and a spot in the FISH analysis was shown in the original paper describing this methodology, it would have been helpful to see a comparison (in at least part of the samples) between the copy number measured by FISH and the copy number measured by absolute quantification in real-time RT-PCR.
5. The presented data provide very strong evidence of the heterogeneity of plus- and minus- strand transcription heterogeneity even within a clonal population with the same proviral integration site. This finding suggests (but does not prove) that viral expression, especially from the plus-strand might follow a pulsatile pattern, which would explain the apparent paradox of a strong immune response and low viral expression in vivo. Time-lapse experiments on live cells expressing a fluorescent reporter of Tax activity (e.g. LTR-unstableGFP) would provide formal proof for this key point.

I have read this submission. I believe that I have an appropriate level of expertise to confirm that it is of an acceptable scientific standard.

# Response

**Published:** 2017-11-30

**Authors:** Charles Bangham

**Affiliations:** Department of Immunology, Imperial College London, UK

**Billman et al 2017: response to reviewers’ comments**

***Reviewer 1 (Ciminale)***

*In the present study Billman et al. used single-molecule RNA-FISH to quantify the expression of HTLV-1 transcripts at the single-cell level. This analysis was carried out in five T-cell clones isolated by limiting dilution from peripheral blood of HTLV-1-infected subjects. Results revealed strong heterogeneity in plus- and minus- strand expression of the viral genome both within and between clones, suggesting that viral genes are transcribed in transcriptional bursts. Interestingly, hbz expression was associated with the S and G /M phases of the cell cycle, and was correlated with nuclear volume.*

*In general, this is an excellent study that applies state-of the-art techniques to tackle some key unanswered questions on the regulation of HTLV-1 gene expression and their possible connection with the viral persistence strategies and pathogenic properties in vivo.*

*The study is very thorough and the data presented are solid and convincing. I have a few suggestions that the Authors might want to consider to improve the quality of this paper.*

*1.  It would be useful to indicate of the location of the probes on the scheme of the genome shown in Fig. 1A.*

Thank you for the suggestion - the probe locations have been added to Figure 1A. 

*2.  Most of the Tax probes will hybridize to ALL plus-strand transcripts, including the highly expressed gag/pol, env and p21 mRNAs. Although the authors clearly state that “tax is used as a proxy for plus-strand proviral expression” referring to Tax may be misleading and is an oversimplification that does not fit well such a careful and thorough study. Indicating this signal as “plus-strand” throughout the paper would be more appropriate.*

At the beginning of the Results section we made this point clear by stating ‘In this paper, *tax* is used as a proxy for plus-strand proviral expression: the *tax* target sequence is present in all plus-strand transcripts.’  However, we accept that the reader may overlook this important point when reading the rest of the paper, and accordingly we have systematically replaced reference ‘ *tax’* expression by ‘plus-strand’ expression in the revised manuscript. 

*3.   Immunofluorescence images should also show the individual channels, not just the overlay.*

We show only the overlay in the main paper for clarity and brevity; a sample of the data is available online, and the full dataset (~2 Tb) is available from the authors on request.

*4.   Although the association between a single mRNA molecule and a spot in the FISH analysis was shown in the original paper describing this methodology, it would have been helpful to see a comparison (in at least part of the samples) between the copy number measured by FISH and the copy number measured by absolute quantification in real-time RT-PCR.*

We agree that it might be helpful to examine formally the correlation between mRNA levels detected by the two techniques (smFISH and qRT-PCR).  However, the results in the present study are corroborated by positive and negative controls, and the addition of qRT-PCR results is unlikely to extend or alter the conclusions reached here.

*5.  The presented data provide very strong evidence of the heterogeneity of plus- and minus- strand transcription heterogeneity even within a clonal population with the same proviral integration site. This finding suggests (but does not prove) that viral expression, especially from the plus-strand might follow a pulsatile pattern, which would explain the apparent paradox of a strong immune response and low viral expression in vivo. Time-lapse experiments on live cells expressing a fluorescent reporter of Tax activity (e.g. LTR-unstableGFP) would provide formal proof for this key point.*

Thank you for this interesting suggestion.  We are in fact already pursuing this approach.

**Reviewer 2 (Bazarbachi/El Hajj)**

*This is a well-written report from leading investigators in the field of HTLV-I replication. In this study, the authors screened more than 19 000 cells derived from five naturally infected T cell clones isolated from PBMCs of HTLV-1 healthy donors. They used single-molecule RNA FISH to quantify, at the single cell level, the transcripts of two main products of the HTLV-I pX region, Tax encoded by the plus-strand of the provirus and HBZ encoded by its minus-strand. They observed a strong intra- and inter-clonal heterogeneity in the expression of tax and hbz genes with Tax being expressed at high levels in few cells whereas hbz is expressed at lower levels in most cells. They also report that both genes are transcribed in intermittent bursts and that tax expression is enhanced in the absence of HBZ but that hbz expression is enhanced by Tax. Finally, they show that HBZ expression is mostly associated with G2/M phases of the cell cycle, and that its abundance correlates with the nuclear volume. They conclude that the main function of this frequent low level of hbz transcript and protein is to maintain clonal persistence and that conversely, intense transient intermittent bursts of tax drive viral replication and clonal proliferation while escaping the robust anti Tax CTL response. These results strongly suggest that the plus strand of HTLV-I is expressed in bursts in vivo explaining the hitherto unexplained paradoxical observations of a strong anti-Tax immune response in all immuno-competent HTLV-I infected individuals while Tax is undetectable in their freshly isolated PBMCs. This is an important study in the field that defeats multiple previous paradigms. The following experiments should strengthen the manuscript:*

*1.   Authors used short-term cultures of activated cells in order to isolate individual cells. However, they need to show tax and hbz expression at the single cell level in freshly isolated PBMCs since cell culture and activation are likely to affect tax and hbz expression.*

We agree that it is of great importance and interest to quantify HTLV-1 expression in freshly isolated PBMCs.  To do this, we are indeed now applying the smFISH technique described here to fresh PBMCs.

*2.   Figure 1: It is surprising that 1% of uninfected clones (negative control) express 50 tax transcripts and only 1 hbz transcript. More uninfected clones should be tested. Furthermore, in order to validate tax and hbz probes, they show the specificity/sensitivity of smFISH on one clone generated from HTLV-I infected cell lines like having different expression levels of Tax and HBZ.*

We apologize for the confusion. In fact, the frequency of cells with > 1 candidate spot in the negative control cells was ~0.6%. The legend to Figure 5 has now been modified to clarify this point.

*3.    It is important to show the viral load and the status of expression of Tax and HBZ in total PBMCs of the 5 HTLV-I positive donors, and compare it with that of the 5 T cell clones that were selected. How can the authors be sure that the selected clone from each donor is a representative one of all the HTLV-1 infected cells in that individual? Did they screen other clones from each of the five donors?*

We have previously shown that a typical HTLV-1-infected host carries > 10 ^5^ infected T-cell clones, it is therefore not practical to study a representative sample of individual clones.  Rather, the present study was designed to establish points of principle. We aim to tackle the question of representativeness by quantifying mRNA expression in fresh polyclonal PBMCs isolated from infected individuals: this work is now in progress.

*4.   Figure 2: Images of tax bursts are more convincing that those of hbz. Figure 2D shows that the estimated number of hbz RNA per burst is around 3 to 4 with little intercellular variation, which is not classical for bursts. How can the authors rule out the possibility that these represent superposition of multiple transcripts. In 2C, they show a three-fold reduction in the frequency of hbz bursts in the presence of actinomycine D compared to a total disappearance of tax bursts. What is the percentage reduction of hbz bursts relative to the reduction of total hbz transcripts?*

Since the abundance of hbz mRNA is so low under all circumstances, there is indeed no strong distinction in the majority of cells between continuous hbz transcription and hbz bursts. The approach we have taken is described in detail in the Materials and Methods section.

*5.   Figure 3: Estimation of intranuclear mRNA should be done in zStacks to rule out counting transcripts above and below the nucleus.*

In developing the current protocol, we did indeed quantify the transcripts in z-stacks lying above or below the nucleus in individual cells. It was not practicable to inspect individual z-stacks on the large total number of cells studied (N > 19,000). However, the small error introduced by quantifying the distribution in 2D would not alter the major conclusions reached here on the distinction between the distribution of plus-strand transcripts and *hbz *transcripts.

*6.    Figure 4: Authors show increased frequency of hbz bursts in high tax positive cells. Again, what about the total amount of hbz transcripts in these cells?*

The total *hbz* spot count is indeed raised in the cells with a high plus-strand spot count.  However, the logistic regression analysis (see Supplementary Figure 10), showed that the *hbz* spot count was correlated with the occurrence of *hbz* bursts, so it may not be meaningful to separate the two parameters here.

*7.    Elevated tax and hbz expression in S and G2/M phase of the cell cycle is solely based on the integrated intensity of the DAPI stained nucleus (2n versus 4n). It would be nice to perform synchronization of cells to confirm these findings.*

This is a good suggestion, and in fact these experiments are already underway.

*8.    Supplementary Figure 9: The negative control (uninfected cells) and clone E should be shown.*

Since the frequency of cells in the negative (uninfected) control (see point 2 above) and the tax-negative Clone E was <1%, the cell-cycle analysis of the frequency distribution of smFISH spots was not carried out with these clones.

*9.    Supplementary Figure 10: The odds ratio for G2/M phase is much higher for cells with >4 hbz compared to cells with >99 tax whereas that for S phase is quite similar. Does this indicate that hbz expression results in G2/M arrest? Is there any correlation between G2/M phase and nuclear size in these cells?*

It has previously been shown that hbz promotes progression through the cell cycle (Satou et al 2006; Mitobe et al 2015: refs. 39 and 40 in the paper). We therefore infer that this is the cause of the high odds ratio referred to, although our results do not exclude the possibility that a proportion of these cells are in G2/M arrest.

**Reviewer 3 (Sitbon)**

*Billman et al. report here a detailed analysis of plus-strand (+strand) "tax" and anti-sense (hbz) RNA transcripts in HTLV-1 T cell clones that have been cultured ex vivo from CD4+CD25+ T cells isolated from PBMCs of 4 different patients. Each clone, harboring a single entire copy of the HTLV-1 genome, was cultured under periodic activation with IL-2, and anti CD2, CD3 and CD28 mAbs. All analyses were performed at day 8 of the 14 day activation cycle, when most cells are returning to a resting state. Single cell analyses of HTLV-1 RNA expression were based on integration of single molecule RNA-FISH and DAPI nuclear staining with occasional actinomycin D treatment.*

*Under these conditions, the authors observed that the vast majority of cells harbored detectable levels of hbz transcripts, while accumulation of +strand transcripts (which they refer to as "tax", see below) was more heterogeneous, varying from <10% in cells from 2 of the clones, to 50-60% of cells from the other 2 clones. An additional clone that harbored a deleted provirus, and thereby did not produce +strand transcripts, served as control. The authors further showed that both sense and anti-sense transcripts could be detected within larger nuclear spots in 4 to 7% of the cells analyzed, which the authors interpreted as transcriptional bursts; this interpretation was confirmed by the significant drop of bursts observed after treatment with actinomycin D. Act D further allowed the authors to show that +strand "tax" transcripts were rapidly accumulating to the cytoplasm while hbz transcripts remained in the nucleus. According to the authors, and in agreement with former observations by several teams, hbz and tax transcripts were in general inversely related. Also, the authors report that both types of transcripts tended to accumulate in cells in G2/M as compared to G0/G1 (monitored as a function of DAPI signal).*

*Elucidation of the in vivo dynamics of Hbz versus Tax production at the single cell level should indeed help elucidate the in vivo regulation of latent versus productive HTLV-1 infection, and provide precious clues on the pathogenic outcomes following infection. Despite an impressive amount of data cross-analyses based on +strand and anti-sense transcription of T cell clones derived from patients' PBMCs, the actual relevance of the model for in vivo situations is questionable. Thus, all analyses were performed on 4 clones isolated from a subpopulation of CD4+CD25+ T cells (as described in a reference and not mentioned here), which after being isolated from patients PBMCs, were cultured for a long period of time in conditions that do not reproduce in vivo conditions (hyperoxia, hyperglycemia, strong recurrent activations and absence of immune pressure). As such, several of the authors' conclusions (in the title, abstract and discussion) are not warranted (see below).*

**

*We agree that it remains to be proved how well the pattern of HTLV-1 transcription we report in these clones in vitro reflects the behaviour of the virus in vivo.  In the paper we do not of course claim to have provided this proof: rather, we state ‘If this pattern of HTLV-1 gene expression accurately represents the pattern of expression in vivo, the paradoxes [three identified] can be explained as follows:’;   and elsewhere ‘The results reported here offer an explanation for each of these paradoxes’ and that our results ‘strongly suggest[s] that the HTLV-1 plus-strand is expressed in bursts in vivo.’  That is, we believe these results offer, but do not prove, a simple and logical explanation of the observations in vivo.  In the revised manuscript we have modified the conclusion section of the Abstract, and added a section to the Discussion (see point 7 below), to further clarify this important point:*

**

*As presented, the study brings up other several additional points:*

*1.   The authors state that all HTLV-1 +strand transcripts are detected with the so-called "tax" probe. Nevertheless, the constant designation of the transcripts as "tax" throughout the article can be very misleading. It would be more accurate to refer to the results obtained with this probe in the text and figures as "+strand", or another more inclusive designation, rather than "tax" solely.*

At the beginning of the Results section we made this point clear by stating ‘In this paper, *tax* is used as a proxy for plus-strand proviral expression: the *tax* target sequence is present in all plus-strand transcripts.’  However, we accept that the reader may overlook this important point when reading the rest of the paper, and accordingly we have systematically replaced reference ‘ *tax’* expression by ‘plus-strand’ expression in the revised manuscript. 

*2.    Nearly 60 to 90% of cells in all 5 clones express hbz antisense transcripts, but depending on the clone studied, the presence of +strand transcripts, as detected with the "tax" probe, are much more heterogeneous (<10% in clones A and D, and 50-60% in clones B & C). Given this overwhelming heterogeneity, how can the authors exclude that it is not due to variation in Tax-encoding transcripts, but solely to other viral +strand transcripts. In other words, the authors cannot exclude that levels of Tax-encoding transcripts were homogeneously low in all clones.*

We have addressed this point in the revised manuscript by replacing ‘ *tax’ *with ‘plus-strand’ (see point 1 above).  In fact, while there is a theoretical possibility that plus-strand transcripts other than *tax* dominate, it is well established that *tax *is the first plus-strand transcript, is most abundant, and crucially *tax *expression is required for efficient expression of other plus-strand RNAs.  Also, even if *tax* were uniformly low in the plus-strand-expressing cells, the single-cell heterogeneity of *tax* would still be much more heterogeneous than that of *hbz.*

*3.   Although the authors insist on the pitfalls of qPCR to analyze single cell transcription, there have been several reports on microfluidics technology being successfully used to monitor single cell RNA expression (i.e. Moussy et al. 2017. PLoS  Biol 15(7):e2001867). The application of microfluidics on PBMCs directly isolated from patients would likely allow a more accurate assessment of the in vivo status of RNA transcripts.*

We agree that it is of great importance and interest to quantify HTLV-1 expression in freshly isolated PBMCs. To do this, we are now applying the smFISH technique described here to fresh PBMCs.

*4.   As mentioned above, given the experimental model of subcloning and culture (at the non-physiological 20% oxygen tension, which artificially modulates HTLV-1 activation, as previously published by the authors), and in the absence of immune pressure, the claim that the results presented here are relevant for in vivo situations and "offer an explanation for the paradoxical correlations observed between the host immune response and HTLV-1 transcription" is not warranted.*

Our recently published evidence (Kulkarni et al 2017, Cell Chemical Biology **24**, 1–11) showed that the oxygen tension modulated plus-strand HTLV-1 transcription, but this effect was approximately two orders of magnitude smaller than the variation in plus-strand abundance reported here, and there was no evidence that oxygen tension initiated plus-strand expression.

*5.   Since the correlation between "transcription bursts" and the G2/M cell cycle stage is not established for +strand "tax" transcripts, and pertains to <7% of cells in which hbz is detected, the claim of a relationship between viral gene bursts and cell cycle (as stated in the general conclusion and the title) does not appear justified. Therefore, the intricate array of cross analyses and statistical evaluation performed here and addressed lengthily in the discussion is not likely to reflect in vivo situations.*

The statistical analysis provides objective evidence of an association between transcription and cell cycle stage, and further shows that this association is independent of the other factors considered.

*6.    Statistical significance of the correlation between high hbz expression, hbz bursts and the G2/M stages should be directly mentioned in Fig. 5 and the corresponding text. The clones used to obtain the data provided in panel 5b are not indicated (it appears that clones A and B were used here, whereas data in 5c are mentioned in the figure legend to result from the average of clones A, B, C and D).*

Thank you for this useful suggestion. In the revised manuscript we have included a reference to the statistical test and significance of the association in the legend to Figure 5; it is also mentioned in the text of the Results section. The cells used in these experiments were the four *tax*-competent clones: this was described in the figure legend; the legend has been modified to make it clearer.

*7.   Given the reservations mentioned above (an in vitro system that does not recapitulate in vivo conditions, the lack of distinction between tax versus +strand transcripts, and the lack of data on Tat and HBZ proteins), the discussion remains too speculative.*

As noted above, we stated in the manuscript ‘If this pattern of HTLV-1 gene expression accurately represents the pattern of expression *in vivo* [...]’ and that our results ‘offer an explanation’ of certain observations *in vivo*. However, we agree that it is important to remind the reader of the actual and potential differences in HTLV-1 transcription between *in vivo* and *in vitro*, and accordingly we have added the following sentence to the end of the Discussion, to make clear what remains hypothetical: ‘It remains to be tested how closely the transcriptional behaviour of HTLV-1 observed in these naturally-infected T-cell clones represents the transcriptional behaviour of the virus *in vivo*, both qualitatively and quantitatively. We are now applying the techniques described here to quantify HTLV-1 plus- and minus-strand transcription in PBMCs both directly *ex vivo *and after short-term *in vitro* incubation.’ We have also modified the concluding section of the abstract, to say ‘If so [if the *in vitro *behaviour accurately represents the *in vivo* behaviour], then our results offer an explanation...’

**

*8.    In this study, there is no assessment of the relationship between transcript and protein levels; moreover, since the half-lives of Tax and Hbz proteins in this model are not established, it is not possible to evaluate the actual timing of a potential cross-regulation between the 2 proteins. In this regard, the authors should discuss a recent paper by Baratella et al. (PLos Negl Trop Dis Jan 2017) that describes the specific accumulation of the HBZ protein in the cytoplasm of PBMCs directly isolated from TSP/HAM patients.*

Thank you for drawing our attention to this interesting paper by Baratella et al. In the revised manuscript we have included the following sentence in the Discussion section to refer to this work: ‘HBZ protein is frequently undetectable in fresh PBMCs, but Baratella et al. ^46^ reported the detection of HBZ protein in the cytoplasm of PBMCs isolated from patients with the inflammatory disease HAM/TSP.’  A detailed examination of the relationship between mRNA and protein levels is beyond the scope of the present paper. 

*9.    In suppl. fig 7 - there is an SEM indicated in the histogram of clone C while there was only 2 replicates, according to the figure legend. Also, in suppl. Figs 8 and 9 there appears to be a color and legend inversion for clones B, C, and D, and clone A does not seem to fit the G2/M correlation with high levels of +strand "tax" transcripts.*

The standard error of the mean is defined for N > 1. Thank you for pointing out the inversions of colour and legend in Figures 8 and 9: these have now been corrected.  Indeed, Clone A did not fit the G2/M correlation with a high frequency of plus-strand transcripts: this point is discussed in the legend to Supplementary Figure 9.
